# Comparing beliefs in falsehoods based on satiric and non-satiric news

**DOI:** 10.1371/journal.pone.0278639

**Published:** 2023-01-19

**Authors:** Shannon H. Poulsen, Robert M. Bond, R. Kelly Garrett

**Affiliations:** School of Communication, The Ohio State University, Columbus, Ohio, United States of America; University of Padova: Universita degli Studi di Padova, ITALY

## Abstract

This article seeks to quantify the extent to which Americans hold beliefs that are consistent with interpreting satiric news literally, and to assess whether factors known to promote misperceptions work differently depending on whether the source of the misperception is satire. We also test the robustness of those factors across a diverse set of real-world falsehoods. The study uses secondary data analysis, relying on data drawn from a 12-wave six-month panel conducted in 2019. Analyses focus on participants’ beliefs about 120 falsehoods derived from high-profile political content in circulation before each survey wave, including 48 based on satiric news. A non-trivial number of participants believed claims originating in satire, but it is less than the proportion who believed falsehoods derived from other misleading content. Results also confirm the robustness of established predictors of misperceptions while demonstrating that the associations differ in magnitude between satiric and non-satiric news.

## Introduction

Unlike many other types of political falsehoods, political satire is not intended to mislead. Instead, it aims to ridicule political subjects, typically relying on irony and exaggeration to critique its target [1]. Yet sometimes people interpret satire literally, leading them to hold misperceptions [2]. This paper examines the extent to which Americans believe falsehoods that originate in news satire. Further, given that satire is different from other forms of misinformation in its intentions, its use of humor, and its themes [3], what is currently understood about misperceptions may not accurately explain how people come to believe claims that originate in satire. This potential gap is problematic—understanding how misperceptions form tells us the most effective ways to reduce their impact. And the notable circulation of satire on social media (e.g., as of June 2022, *The Babylon Bee* had 1.5 million followers on Twitter) may make misperceptions based on it more prevalent, increasing the importance of understanding how to best reduce them. Thus, we also assess whether falsehoods that originated in online satiric news are believed at a different rate than those promoted in other types of misleading online content, and we test a handful of mechanisms that may explain such differences.

The data used to assess these questions are drawn from a larger project designed to examine Americans’ beliefs in political claims, both true and false, that were widely shared on social media over a six-month period. We did not set out to study satire; instead, the importance of satire in the social media ecosystem emerged organically from the data we collected. We capitalize on the fact that satiric news was often found among the most widely shared falsehoods during data collection in early 2019 to assess whether belief in claims originating in satire differ from beliefs in falsehoods from other sources in significant ways.

Importantly, we did not show participants satire. Instead, we asked people about their beliefs about false claims made in satire and elsewhere that were already widely shared on social media at the time of the study. Although there is value in understanding how people assess satiric news upon exposure, our approach has several benefits. First, it allows us to compare beliefs resulting from literal interpretations of real-world satire to beliefs about other types of falsehoods. Second, our approach helps us to understand satire’s indirect consequences, assessing its influence on both those who have direct contact with the claims from satiric sources and those who encounter those falsehoods in other contexts (e.g., when a trusted source repeats the claim as though it were true).

## Background

Accurate political knowledge is fundamental to democracy [4]. When someone believes something that is not consistent with the best available evidence, they hold a misperception. Misperceptions can be the byproduct of memory failures and biased interpretations [5, 6], but they are also often the result of believing misleading messages [7]. One widely cited typology of misinformation identifies seven different types, loosely organized in terms of their “intent to deceive” [8]. One end of the spectrum consists of content that is fabricated with an explicit intention to mislead and do harm (“disinformation”). The other end of the spectrum consists of satiric news (sometimes referred to as news satire or spoof news). Satiric news seeks to entertain audiences by humorously parodying traditional news while criticizing politics, politicians, and the broader society [9]. In this pursuit, satire distorts reality and thus offers information that, if taken literally, is false.

Importantly, satiric news is not designed to mislead; in some cases, it can be informative. For instance, consuming long-form satiric television news programs, such as *The Daily Show* and *Last Week Tonight*, is associated with increases in political knowledge [10]. These benefits, however, are typically associated with instances in which humor succeeds, that is, when the audience recognizes and understands the humor, and finds the message to be funny. In such cases, satire can be a democratic good.

There is, however, no question that people are sometimes fooled by satiric news [11, 12]. In these instances, the attempt at communicating a message via humor has failed [13] and as a result, people interpret the satiric claims literally. When this happens, people can form satire-induced misperceptions. In this study, we focus on beliefs in claims that originate in satiric news articles, a text-based form of satire that mimics hard news journalism. Despite anecdotal evidence that literal interpretations of satire are widespread, there is little empirical work quantifying how often Americans’ beliefs reflect exaggerations promoted in satire. This is a consequential oversight, as satiric news is fundamentally different from other types of mis- or disinformation, suggesting that beliefs, and how they are formed, may also be different. In the section below, we describe several differences between satire and non-satiric misinformation.

### Satire is a different kind of misinformation

The most notable difference between satire and other sources of misinformation is that satire distorts its claims to be funny. Satire’s features can make the falsehood easier to detect but sometimes may have the opposite effect. We first review scholarship that helps explain why people are more likely believe literal interpretations of satire than to hold misperceptions based on other types of content. Then, we turn to scholarship describing how use of humor can promote accuracy. Note that we do not test these mechanisms in this study; instead, we offer them as background for our predictions that beliefs based on satiric news and other sources of false information will differ.

The presence of humor can make satiric claims more likely to be believed. Understanding satire is a complex process, requiring both knowledge of relevant political topics and cognitive effort [14]. Recipients who are unfamiliar with the events being satirized are unlikely to detect factual inconsistencies in a satiric retelling, as are those who are unwilling to exert effort to make sense of any inconsistencies that they detect [15]. Even if people do invest the effort, satiric content is often intentionally ambiguous, which can encourage selective, and often partisan, interpretations. Most people dislike seeing their ingroup ridiculed, and one way to avoid this unpleasant experience is to misinterpret satire as an endorsement of the satirized position. This may help to explain, for example, why many conservative viewers of the *Colbert Report* misunderstood Stephen Colbert’s satiric portrayal of a conservative pundit, believing him to be a conservative who mocked liberals, rather than a liberal mocking conservatives [12].

Understanding humor used in satire, and therefore disbelieving the literal claims it makes, is also likely influenced by the format of satiric news. Text-based satiric news articles have fewer humor markers—message characteristics that signal their humorous intent—compared to other types of satire [16]. For example, satiric news videos often include audience laughter that calls attention to the punchline. Such markers are absent from satiric news articles.

Satire may also be easier to mistake for news given the political climate in the U.S. Americans live in an environment in which political elites regularly engage in actions that would have violated expectations of political behavior just a short time ago. For example, the *Washington Post* estimates that while serving as president, Donald Trump made over thirty thousand false or misleading claims [17]. Furthermore, expressions of outrage about “egregious” actions taken by the political opposition are widespread on both sides of the political aisle [18]. In the contemporary political environment, outrageous claims may appear plausible to many. In short, it is hard to understand satire—whether it be due to a lack of knowledge, lack of effort, biased interpretations, or normalized egregious behavior—which may make people more susceptible to holding satire-induced misperceptions than other types of misperceptions.

It is, however, also possible that the use of humor may make satiric claims more likely to be dismissed than other claims. Satire features more hyperbolic claims than other forms of misinformation [3], and these claims are often absurdly distorted—no one would be expected to believe that Joe Biden spoke in tongues at a campaign event for Pentecostals, or that President Trump punched a baby because he thought it was a member of Antifa. (Both claims were made in satiric news stories published during the 2020 U.S. Presidential election). Such absurdity likely suggests to readers that the claims are untrue. Furthermore, many publishers of satiric news include disclaimers about the fictious nature of their content on their websites, and some are at least modestly well known (e.g., *The Onion*).

It is also often harder to recognize other types of mis- and disinformation than to recognize satire. Hyper-partisan news outlets and sources of foreign propaganda regularly make inaccurate claims that lack obvious cues about their questionable nature [19]. In short, both the presence of hyperbolic claims in satire and the inherent difficulties with recognizing mis/disinformation may mean people believe claims derived from satire less than claims derived from other forms of misinformation.

Whether the presence of humor aids in the rejection of satire-induced misperceptions, or promotes them, there are likely to be differences in how often people fall for satire versus other misinformation. This leads us to the following hypothesis: Misperceptions will be held at different rates depending on whether the origin of the claim is satiric news or non-satiric sources of misinformation (H1).

### Factors that influence misperceptions

Next, we summarize factors shown to promote or constrain misperceptions in the extant literature, and we argue these mechanisms may work differently when people are forming satire-induced misperceptions.

Demographic characteristics, psychological attributes, news consumption practices, and social media factors have all been linked with belief in falsehoods. For example, individuals who have more education or who are more politically interested tend to be more politically knowledgeable [20], suggesting that they might also be less likely to hold misperceptions. Men also tend to score higher on tests of political knowledge [20]. Furthermore, people tend to be predisposed to believe claims that are good for their political ingroup and to disbelieve those that threaten the ingroup [21, 22]. There are also differences associated with ideology. For example, older, more conservative Americans are uniquely likely to engage with false information shared online [23] which could indicate higher levels of belief in misinformation. Recent scholarship suggests that, in the U.S. at least, high-profile falsehoods are more likely to benefit conservatives than liberals, leading those identifying with the former group to hold relatively more misperceptions [24]. Individuals’ epistemic beliefs—beliefs about the nature of knowledge—are also predictive of political beliefs. The more faith individuals put in their ability to intuitively know what is true, and the more they view truth as a political construct, the more likely they are to label falsehoods as true. Conversely, the more value an individual places on empirical evidence, the more accurate they tend to be [25]. Conspiracist thinking is also associated with misperceptions that go beyond conspiracy theories [26].

Media use can also be influential. Under the right conditions, news use has been shown to promote political knowledge [27] which should enhance people’s ability to recognize falsehoods. There is more uncertainty about how use of social media might shape belief accuracy. The wide spread of falsehoods on social media platforms suggests that these technologies could promote misperceptions [28, 29]. However, most American social media users have seen relatively few falsehoods, and the relationship between the frequency of social media use and misperceptions appears weak or non-existent [7, 30]. If social media has any influence on misperceptions, it is more likely to affect users’ beliefs about the most viral falsehoods. We have long understood that more frequent exposure to a message is associated with greater belief [31] and social media magnifies attention to a small subset of content. For false content, high levels of social media engagement could promote misperceptions.

### Do misperceptions vary depending on their origin?

The central question of this paper is whether belief in falsehoods embedded in satiric news (e.g., exaggerated descriptions of politicians’ behaviors that were, in their original context, intended to be funny) is promoted or inhibited distinctly from belief in other falsehoods (e.g., misleading claims about politicians’ behavior that were never meant to be funny). As a reminder, our focus is on belief in the false claims promote by a satiric story, not the satiric headline itself. In other words, this work is not about satire recognition but about belief in falsehoods that originate in satire. There is relatively little work in this domain, so our predictions are necessarily speculative.

We do not expect differences in every case—for example, education is equally likely to promote accuracy when assessing satiric and non-satiric falsehoods—but there are reasons to suspect that many predictors’ influence is contingent on misinformation type. We organize the following arguments in four categories: demographic characteristics, psychological attributes, news consumption practices, and social media factors.

Demographic differences may affect misperceptions differently depending on their origin. For example, younger people are more likely to get absorbed in satire, investing more cognitive resources into processing the message and less into thinking about it critically [14, 32]. It is possible, then, that younger people will be more likely to be misled by satire. Will the relationship between age and holding misperceptions differ depending on the origin of the misperception (RQ1)?

Implications for one’s social identity may also affect belief in claims originating in satire in distinct ways. Recall that claims may harm the ingroup, benefit the ingroup, or have no effect on the ingroup. We focus here on the first two types. People may be more likely to believe claims based on satire that harm the ingroup—satire is often ambiguous, and the recipient may mistakenly believe that a harmful satirical claim promotes their ingroup, which would predispose them to believing it. In other cases, people may be more likely to reject harmful claims made in satiric news. Funny messages are more likely to be dismissed as “just a joke” [33] which could be appealing if the satiric news headline is critical of the ingroup. For claims that benefit the ingroup, people who are otherwise predisposed to believe the claims may be more skeptical when they originate in satire. The hyperbolic nature of these claims may make it harder to justify interpreting the claims in a biased manner. The bottom line, though, is that partisans may react to falsehoods with ingroup implications and based on satire differently than falsehoods with comparable ingroup implications but based on other misleading content. Will the relationship between whom a claim harms and holding misperceptions differ depending on the origin of the misperception (RQ2a)? And will the relationship between whom a claim benefits and holding misperceptions differ depending on the origin of the misperception (RQ2b)?

Political ideology or party affiliation might also work differently when predicting belief in satire than other falsehoods. Conservatives (i.e., Republicans in the U.S.) are more prone to endorsing misperceptions than liberals (i.e., Democrats in the U.S.) even after accounting for the social identity implications of misinformation [24]. Yet there are reasons to doubt this party difference will persist when it comes to claims from satire. Liberals and conservatives understand different types of humor at similar rates [34], suggesting that both parties will be equally equipped to reject claims found in satire. Will the relationship between party identification and holding misperceptions differ depending on the origin of the misperception (RQ3)?

The influence of several psychological factors might also be contingent on whether the falsehood is based on satiric news. For example, although individuals who rely more heavily on intuition tend to hold more misperceptions, that same intuition may help them better recognize incongruities in humor [35]. It also could be that those who put more value on evidence when forming their beliefs might be less likely to mistake satire for real news precisely because satire lacks a secure standard of evidence. Individuals who think that truth is politically constructed (that is, there is no objective truth) may be less likely to use that epistemic belief to justify describing a claim originating in satire as true. This style of argumentation is effortful and, as such, it may be reserved for more plausible claims. Will the relationship between one’s (RQ4) faith in intuition, (RQ5) need for evidence, (RQ6) or the perception that truth is political and holding misperceptions differ depending on the origin of the misperception?

As for conspiracy thinking, the propensity to believe in secret organizations and networks of coordinated activity could be an indication of general credulity [36], in which case satiric claims are as likely to be believed as any other falsehood. Compared to other types of misleading news content, however, satire is less likely to explicitly feature conspiracy theories [3]. This suggests that conspiracy thinking may be a weaker predictor of belief in satire than other falsehoods. Will the relationship between conspiratorial thinking and holding misperceptions differ depending on the origin of the falsehood (RQ7)?

We also might expect the accuracy-inducing effects of news consumption to be larger when assessing claims made in satiric news. The more people know about the political events of the day (via news exposure), the more likely they are to notice the ways in which satiric coverage deviates from other accounts, which increases their chances of recognizing humor as the explanation for the deviation [35]. Perhaps less intuitively, though, news use could also have a harmful effect. Satiric news parodies the format, tone, and style of traditional news [9] and those who consume news more frequently may be more likely to be misled when these cues appear in satiric news. Will the relationship between (RQ8a) online news consumption or (RQ8b) offline news consumption and holding misperceptions differ depending on the origin of the falsehood?

Finally, we consider whether social media factors have more influence when a story is satiric than when it is not. One factor is social media use. Satiric posts are designed to mimic the appearance of other social media news content [9]. Perhaps increased use of social media normalizes the appearance of news satire, suggesting the content is true. At the same time, increased social media use might give people more practice at recognizing satire, decreasing the likelihood of belief. Will the relationship between social media use and holding misperceptions differ depending on the origin of the misperception (RQ9)?

Social media engagement may also influence satire-induced misperceptions. One troubling possibility is that widespread sharing of a satiric news story could overcome the skepticism that most individuals exhibit toward satire via the illusory truth effect [31]. That is, perhaps beliefs in claims based on satire and in claims based on other misleading content will be more similar the more engagement a false story receives. It is also possible that increased engagement may signal credibility via a bandwagon heuristic [37]. Will the relationship between story-engagement and holding misperceptions differ depending on the origin of the misperception (RQ10)?

In sum, we expect there to be differences in misperception endorsement based their origin. We also expect that some factors known to promote misperceptions may have a unique influence when people are assessing claims derived from satire.

## Materials and methods

Original data were collected as part of a large panel study designed to examine a series of questions related to political misperceptions [24]. This project is a secondary analysis of that data. The original project was approved by the [redacted] Institutional Review Board (Study number [redacted]). Consent was given digitally via an online consent form.

As noted in the introduction, this was not designed as a study of satire; instead, satire’s importance only became clear over the course of data collection. The research team contracted YouGov to conduct the panel, surveying a large, demographically diverse sample of Americans every two weeks for six months (baseline *N* = 1,204). Retention between waves was acceptable, varying between 66.5% and 75.4% (*n*s = 801–908). Participants were invited to return to the panel after missing a wave, and most (76.1%) completed at least half the waves. Given that one of our goals is to examine potential party differences in belief by falsehood origin, we analyzed responses only from panelists who identified as Democrat or Republican (*n* = 732). We also excluded panelists who failed to provide data for our variables of interest (e.g., conspiratorial thinking, which was only measured in the last wave) and covariates (e.g., education), bringing our final sample size for analyses to 480 panelists.

Our final sample is demographically diverse and broadly reflects the U.S. population. It is racially diverse (76% White, 10% Hispanic, 10% Black), includes a fair age range (9% 18–29, 18% 30–44, 26% 45–59, 47% 60+), a mix of educational attainment (34% High school or less, 34% some college, 32%, Bachelor’s degree or higher), and is fairly politically diverse (55% Democrats, 45% Republicans). Women are somewhat over-represented (56%).

A core component of the questionnaire was a battery of items measuring participants’ beliefs in political claims made in high profile stories recently published online. We used Facebook engagement data (like, shares, and comments) provided by NewsWhip to identify the most engaging web content making political claims including both satiric news and other sources of misinformation. All but two sources were categorized based on MediaBiasFactCheck (MBFC). MBFC defines satirical sources as those that “exclusively use humor, irony, exaggeration, or ridicule to expose and criticize people’s stupidity or vices… these sources are clear that they are satire and do not attempt to deceive” [38]. We reviewed how the two remaining sites described themselves and classified accordingly. We excluded content that was not related to U.S. politics or that had ambiguous veracity. Finally, we selected the ten most engaging true and false stories from those that remained and wrote short statements summarizing key claims made explicitly by each.

It is important to note that this process is different than asking participants about the veracity of a story. We instead focused on their belief in claims advanced by these stories. For satire, that means participants evaluated whether they believed claims based on the literal interpretation of satire headlines. For example, the statement “To avoid having to answer questions about her Green New Deal, Rep. Alexandria-Ocasio Cortez claimed to not speak English” was based on the satiric headline, “Confronted With Details Of Green New Deal, Ocasio-Cortez Claims Not To Speak English.” None of the statements were intended to be humorous—not even those based on satiric news. Instead, each statement described a false claim advanced in the story.

This process yielded a total of 120 falsehoods over the course of the study. See Table 1 for story descriptive statistics by origin. Importantly, 48 (40%) of the falsehoods were based on satiric news, distributed roughly evenly over the duration of the panel (see S1, S2 Tables in the S1 Appendix for statement wording and sources). This metric alone demonstrates that satiric news makes up a notable portion of misinformation receiving high levels of engagement. Statements from satiric sources also were more likely to criticize Republicans than those from non-satiric sources. Statements were presented in random order, regardless of their source. We did not show source information, images that accompanied the original news story, etc.; participants saw only the statements based on the stories.

**Table 1:** False statement and story descriptives by message type.

	Satire	Non-satire
**No. of false statements**	*M* = 4 per wave (*SD* = 2)	*M* = 6 per wave (*SD* = 2)
	*n* = 48 total	*n* = 72 total
**No. of unique sources**	14	34
**Political slant** ^**a**^	35.42% favor Democrats	15.28% favor Democrats
	37.50% favor Republicans	51.39% favor Republicans
**Strength of belief**	2.35 (.83)	2.41 (.85)

^a^ Remaining statements were labeled politically neutral

Separately, we used Amazon’s Mechanical Turk to determine the statements’ political slant. We paid ten workers, split evenly between Democrats and Republicans, to label each statement in terms of its implications for their party and the opposition party. If workers belonging to both parties agreed that a statement benefitted one party more than the other, or if workers in one party indicated the statement benefitted one party more while the workers from the other party said the statement was neutral, we labeled it as slanted in favor of that party. If both groups of workers said the statement was neutral or gave contradictory answers, we labeled it neutral. This approach is similar to commonly used practices of classifying content into ideological categories [39].

The study was approved by the [Redacted] Institutional Review Board [study number redacted]. Consent was obtained electronically.

### Measures

The longitudinal design of the study meant that measures varied in terms of when they were collected: once per statement, once per wave, or once per participant. We first describe statement-level measures. Strength of belief was measured on a four-point scale, anchored by definitely false (1) and definitely true (4), and serves as our outcome measure. As noted above, we measured which party would benefit or be harmed by a statement if it had been true using the data collected on MTurk. A statement could be neutral, could benefit the ingroup, or could harm the ingroup. We captured this with a pair of dichotomous variables. If (and only if) a statement benefited the participant’s party affiliation (e.g., a Democrat evaluating a statement favoring Democrats), it was labeled it ingroup beneficial (33.83%). Likewise, if the statement benefited the other party (e.g., a Democrat evaluating a statement favoring Republicans), it was labeled it ingroup harmful (35.78%).

Next, we summarize questions asked once per wave. All types of media use are self-reported on a five-point scale that ranged from “several times a day” (5) to “less often [than every few weeks]” (1). Offline news use is the average of five channels: television news, print newspapers, print news magazines, radio news or talk radio, and television talk shows (*M* = 2.37, *SD* = .95). Online news relies on a single measure to capture use of “online news sites, blogs, or news apps” (*M* = 3.01, *SD* = 1.56). Social media use is the average of self-reported Facebook, Twitter, and Reddit use (*M* = 2.30, *SD* = .92). While there are more social networking sites that may be of interest (including, but not limited to, Instagram and TikTok), our operationalization expands beyond simply Facebook and/or Twitter use to also include Reddit use. Social media engagement is based on the number of Facebook likes, comments or shares that the news story on which a claim was based received according to NewsWhip, a social media monitoring company (Min = 13,450, Max = 2.95M, *M* = 155,493, *SD* = 330,013, Skewness = 6.4, Kurtosis = 50.2). This value is then log transformed.

Finally, we have measures that were captured once per participant. Demographic predictors include age, sex, education, and party affiliation. All measures of psychological attributes are based on established scales: Faith in Intuition for Facts (*M* = 3.39, *SD* = .66), Need for Evidence (*M* = 4.00, SD = .62), Truth is Political (*M* = 2.69, *SD* = .86) [25] and the Conspiracy Mentality Questionnaire (CMQ, *M* = 3.76, *SD* = .66) [40]. CMQ was only measured in the final wave, which means that only those who participated in this wave were included in these analyses. Excluding this item from the models reported below increases the sample size but does not substantively alter patterns of results.

### Analytic approach

Our analyses focus on how individual differences shape beliefs; we do not use the panel design to assess temporal dynamics. Nevertheless, our analytic approach must reflect the complex structure of these data. With almost five hundred participants, each contacted up to twelve times, and each assessing ten falsehoods per wave, we have roughly fifty thousand data points. These observations are clearly non-independent. To assess associations between variables of interest and accuracy of belief, we construct three-level mixed effects regression models, nesting waves within participants and clustering on participant ID.

## Results

**Fig 1:**
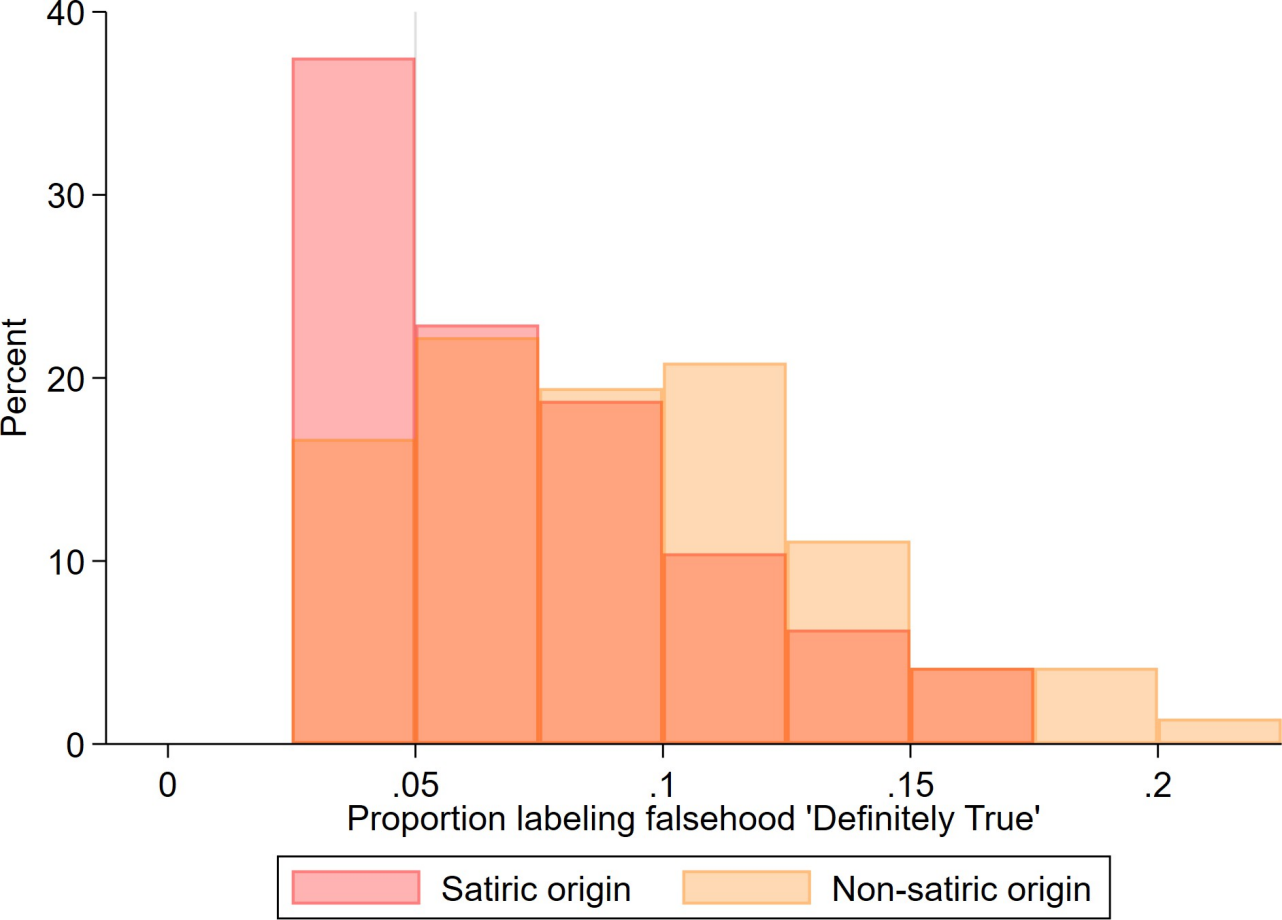
Distribution of strong belief in falsehoods by source. Statements believed by 5% or less (grey vertical line) are unlikely to reflect sincere beliefs. Proportions are weighted.

First, we examine descriptive statistics relevant to our arguments. Overall belief in claims originating in satire was modest. The proportion of participants labeling claims based on satiric news stories as “definitely true” ranges from less than 3% to about 16% (average = 7%) across the 48 satiric claims assessed in this study (and see Fig 1). This is slightly lower than the proportion labeling non-satiric falsehoods in the same way, which ranged from less than 3% to about 20% (average = 9%). We note that statements believed by fewer than 5% of the sample are not necessarily indications of legitimate belief. In most surveys, a small proportion of respondents engage in “expressive responding,” reporting belief in claims simply because those claims are good for their party [41]. In the weeks following the 2020 election, another study we conducted using a design similar to the one used here found that the proportion of participants who labeled false statements constructed by researchers (and thus not actually circulating at the time) as “definitely true” ranged from about 2% to about 5% (average = 4%), which we use as an assessment of the prevalence of expressive responding. Many falsehoods in the present study that were based on satiric news were marked “definitely true” by well more than five percent of the sample, suggesting that a non-trivial number of Americans sincerely believed literal interpretations of at least some satiric news circulating over the six-month study period.

Belief in many of the most prominent satiric claims also varied by party, though this was not always the case (see Table 2).

**Table 2:** Examples of falsehoods based on satiric news.

D%	R%	Statement evaluated by participants	Source headline
9.13%	6.38%	Following the passage of Alabama’s new restrictive abortion bill, a 12-year-old victim of sexual abuse said during an interview that she doesn’t think she can be a mom on top of her already hectic life.	Abused 12-Year-Old Alabama Girl Doesn’t Think She Can Handle Being A Mom on Top of Everything Else (*The Onion*)
6.94%	20.22%	Representative Ilhan Omar said that being Jewish is an inherently hostile act, especially among those living in Israel.	Ilhan Omar: ’If Israel Is So Innocent, Then Why Do They Insist on Being Jews?’ (*The Babylon Bee*)
7.39%	5.85%	Alabama State Senator Katie Shaw proposed an amendment to the state’s new abortion bill requiring that unmarried men get vasectomies that could only be reversed when they marry.	Alabama Democrat Proposes Mandatory Vasectomies Until Marriage (*Alternatively Facts*)

D% and R% are the proportions of Democrats and of Republicans labeling the statement “definitely true.” Sources shown in parenthesis after the source headline.

Turning to our theoretical questions, we first evaluate H1, which predicts that belief in falsehoods will be influenced by its origin. We start wih a mixed effects regression model predicting strength of belief that includes an effect for the type of misinformation as well as main effects for each of the factors known promote misperceptions (see Table 3). In support of H1, we find that there is a signficiant difference in the likelihood of belief in falsehoods depending on their origin. False claims made in satiric stories were believed less often than those from other sources, *b* = -.068, *p* < .001.

**Table 3:** Mixed effects regression model of belief in falsehoods.

Factor	Coefficient	Standard Error	*p*	95% CI
**Satire**	-0.068	0.010	<0.001	-0.089, -0.048
**Education**	-0.025	0.010	0.016	-0.046, -0.005
**Political interest**	-0.076	0.018	<0.001	-0.112, -0.040
**Sex (Male = 1)**	-0.056	0.029	0.059	-0.113, 0.002
**Age**	-0.001	0.001	0.234	-0.003, 0.001
**Party ID (Democract = 1)**	-0.239	0.030	<0.001	-0.298, -0.179
**Ingroup-beneficial statement**	0.103	0.012	<0.001	0.080, 0.126
**Ingroup-harmful statement**	-0.236	0.011	<0.001	-0.258, -0.214
**Faith in intuition for facts**	0.100	0.024	<0.001	0.054, 0.146
**Need for Evidence**	-0.019	0.026	0.463	-0.069, 0.031
**Truth is Political**	0.086	0.020	<0.001	0.046, 0.125
**Conspiracy mentality**	0.148	0.024	<0.001	0.101, 0.195
**Average online news use**	0.004	0.005	0.400	-0.006, 0.015
**Average offline news use**	0.027	0.012	0.022	0.004, 0.049
**Average social media use**	0.024	0.014	0.100	-0.005, 0.052
**Facebook engagement (log)**	0.035	0.004	<0.001	0.028, 0.042
**Intercept**	1.457	0.174	<0.001	1.117, 1.798
**Number of observations**	50926			
**Number of clusters**	480			
**χ²**	1164.626			
**Model test *p*-value**	<0.001			
**AIC**	110432.2			
**BIC**	110608.9			

Before we assess whether factors that promote misperceptions do so differently depending on the source of the claim, we first assess the robustness of these factors for all misperceptions (regardless of source) in the same regression model. Education, *b* = -.025, *p* = .016, and political interest, *b* = -.076, *p* < .001, were associated with lower belief in falsehoods, though neither age nor sex had a significant influence. Consistent with other analyses using these data, party affiliation also had a strong effect: Democrats were significantly less likely than Republicans to believe falsehoods, *b* = -.239, *p* < .001. Unsurprisingly, participants were more likely to believe claims that promoted their party over the opposition, *b* = .103, *p* < .001, compared to those that were politically neutral; and they were less likely to believe claims that devalued their party, *b* = -.236, *p* < .001, compared to those that were politically neutral. Two of the three epistemic beliefs were significant factors in the model. Individuals who put more faith in their ability to intuitively recognize facts, *b* = .100, *p* < .001, and who view facts as politcally constructed, *b* = .086, *p* < .001, were more often inaccurate, though valuing evidence did not have the predicted countervailing effect. Similarly, those who engage in conspiracy thinking are also more likely to be inaccurate, *b* = .148, *p* < .001. Offline news use was associated with believing falsehoods more strongly, *b* = .027, *p* = .022; neither online news use nor social media use was significantly associated. Finally, the level of Facebook engagement with the story containing the falsehood was positively associated with belief, *b* = .035, *p* < .001.

Next, we consider whether these effects are contingent on whether the falsehood was derived from satiric news. These tests extend the prior regression model by adding an interaction between whether the source of the falsehood was satire and the predictor of interest. With ten research questions, two of which contain multiple questions, we estimated twelve additional regression models to evaluate whether story type affects a factor’s relationship with belief in falsehoods. We provide full model estimates in the Supplmentary Information file (See S3 through S14 Tables in S1 Appendix). For ease of interpretation, Tables 4–6 provide the interaction term of interest from its respective regression model and the table number in the supplementary materials. We find that many effects are contingent on whether the source of a falsehood is satiric news.

**Table 4:** Mixed-effects regression models on belief in falsehoods with respective interactions between story type and demographic/identity-based factors.

Interaction term^a^	Coefficient	Standard Error	*p*	95% CI	Table with full model results
**Age x Satire**	-0.001	0.001	0.115	-0.002, 0.000	S3 Table in S1 Appendix
**Claim is ingroup beneficial x Satire**	-0.196	0.015	<0.001	-0.225, -0.167	S4 Table in S1 Appendix
**Claim is ingroup harmful x Satire**	0.006	0.013	0.640	-0.019, 0.031	S5 Table in S1 Appendix
**Party ID x Satire**	0.218	0.017	<0.001	0.184, 0.252	S6 Table in S1 Appendix

^a^ The interaction terms provided here are from separate regression models on belief in falsehoods.

**Table 5:** Mixed-effects regression models on belief in falsehoods with respective interactions between story type and psychological factors.

Interaction term ^a^	Coefficient	Standard Error	*p*	95% CI	Table with full model results
**Faith in intuition x Satire**	-0.074	0.016	<0.001	-0.106, -0.043	Table S7 in S1 Appendix
**Need for evidence x Satire**	0.033	0.017	0.055	-0.001, 0.066	Table S8 in S1 Appendix
**Truth is political x Satire**	-0.055	0.013	<0.001	-0.080, -0.030	Table S9 in S1 Appendix
**Conspiracy mentality x Satire**	-0.059	0.016	<0.001	-0.090, -0.029	Table S10 in S1 Appendix

^a^ The interaction terms provided here are from separate regression models on belief in falsehoods.

**Table 6:** Mixed-effects regression models on belief in falsehoods with respective interactions between story type and news/social media use factors.

Interaction term ^a^	Coefficient	Standard Error	*p*	95% CI	Table with full model results
**Average online news use x Satire**	-0.014	0.006	0.024	-0.025, -0.002	Table S11 in S1 Appendix
**Average offline news use x Satire**	-0.006	0.010	0.563	-0.026, 0.014	Table S12 in S1 Appendix
**Average social media use x Satire**	0.001	0.012	0.898	-0.021, 0.024	Table S13 in S1 Appendix
**Social media engagement x Satire**	0.017	0.007	0.018	0.003, 0.031	Table S14 in S1 Appendix

^a^ The interaction terms provided here are from separate regression models on belief in falsehoods.

**Fig 2:**
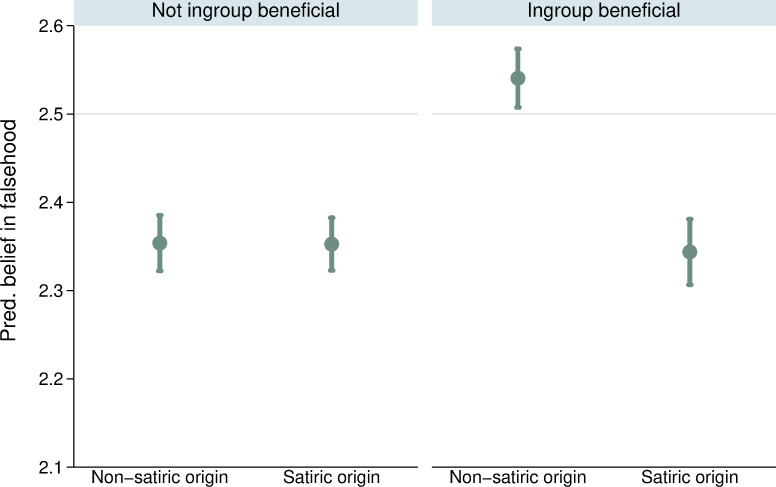
Estimated strength of belief by whether statement benefits political ingroup and source of falsehood. Points correspond to point estimates with lines denoting 95% confidence intervals. Thin grey line indicates scale midpoint.

We start by examining the demographic factors (RQ1-RQ3). We found no evidence that the relationship between age and belief in falsehoods (RQ1) was contingent on whether the source was satiric, *b* = -.001, *p* = 0.115. Other factors are contingent on the origin of the misperception. We find mixed support for social identity biases. Claims that benefit the ingroup (which people are predisposed to believe; RQ2a) are more likely to be rejected when based on satire, *b* = -.196, *p* < 0.001 than when they are based on other sources (see Fig 2). However, we did not find evidence that claims that harm the ingroup (which people are predisposed to reject; RQ2b) are more likely to be rejected when based on satire, *b* = .006, *p* = 0.640.

**Fig 3:**
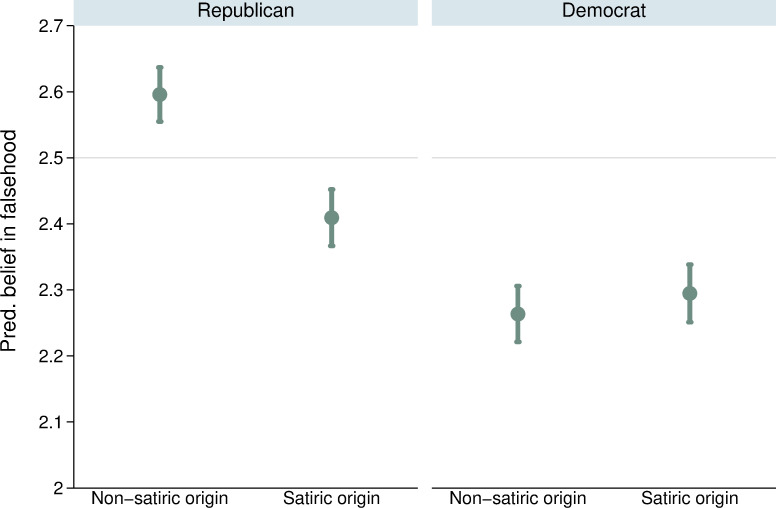
Estimated strength of belief by party identification and source of falsehood. Points correspond to point estimates with lines denoting 95% confidence intervals. Thin grey line indicates scale midpoint.

We also find that the influence of party affiliation (RQ3) varies by the type of falsehood, *b* = .218, *p* < .001. Although Republicans tend to perform worse than Democrats at identifying falsehoods generally, the partisan accuracy gap is smaller when assessing claims originating in satiric news than other types of misinformation (see Fig 3). Republicans are more likely to reject claims originating in satire than other types of falsehoods. We discuss this finding more in the discussion section.

Three psychological factors also had unique relationships with misperceptions depending the origin of the falsehood. Faith in Intuition for Facts (RQ4; *b* = -.074, *p* < 0.001), perceptions that the truth is political (RQ6; *b* = -.055, *p* < 0.001), and conspiracy mentality (RQ7; *b* = -.059, *p* < 0.001) are all associated with stronger belief in falsehoods, but their relationship is attenuated for satirical claims (see S1-S3 Figs in S1 Appendix). Need for evidence did not significantly vary in its effect on belief by misinformation type, *b* = .033, *p* = 0.055.

One type of news consumption is also contingent on the source of the falsehood (RQ8). Although online news use did not have a main effect, it was associated with less belief in satiric claims than in non-satiric claims (*b* = -.014, *p* = 0.006; see S4 Fig in S1 Appendix). We did not find evidence that offline news use’s influence on belief varied by the source of a falsehood, *b* = -.006, *p* = 0.563.

**Fig 4:**
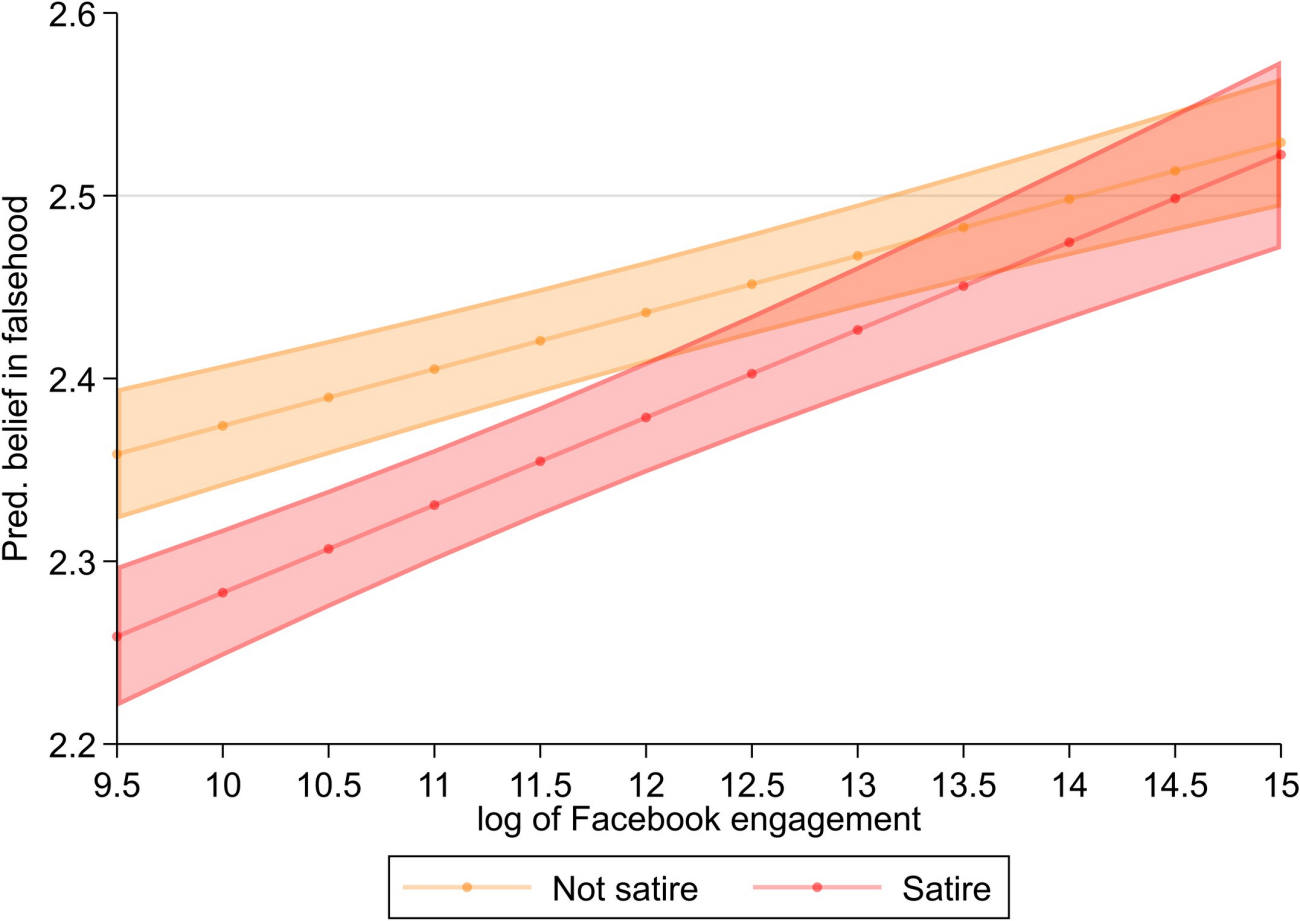
Estimated strength of belief by Facebook engagement and source of falsehood. Lines correspond to point estimates with shaded areas denoting 95% confidence intervals. Thin grey line indicates scale midpoint.

Finally, one social media factor’s influence on misperceptions is contingent on misperception origin (RQ10). The more engagement a false story received on Facebook, the more likely it was to be believed, and this relationship was stronger for satiric news stories, *b* = .017, *p* < .05 (see Fig 4). Importantly, even modest Facebook engagement (i.e., around 50K or higher) offset participants’ tendency to disbelieve satiric stories. We consider the significance of this relationship in the discussion. We failed to find support that social media use promoted belief in satiric claims differently than claims from non-satiric sources, *b* = .001, *p* = 0.898. This may be a result of our operationalization of social media use, which focused on Facebook, Twitter, and Reddit use only.

## Discussion

Misperceptions based on claims originating in satiric news are not as widespread as those based on other sources, but such misperceptions are held by a non-trivial number of Americans. Several of the claims included in this study were described as “definitely true” by upwards of 10% of our sample. Although some claims of belief are probably insincere, serving more as signals of partisan identity than as legitimate assessments of veracity, the levels of self-reported belief in many satiric claims are considerably higher than the small proportion of Americans who regularly engage in “partisan cheerleading” in surveys [41]. In short, many people believe at least some false claims shared in political satire. This is, to our knowledge, the first study to use a wide range of real-world political content to quantitatively assess the extent to which people hold beliefs that are consistent with a literal interpretation of satiric news stories.

Turning to possible mechanisms for explaining this difference, we show that several factors’ associations with misperceptions are contingent on whether the misleading claim was circulated via satiric news. We observed differences in the influence of social-identity implications of the falsehoods, party affiliation, participants’ faith in the intuition for truth, perceptions that the truth is political, conspiracy mentality, online news use, and Facebook engagement. In this discussion we focus on three important results, starting with the one we consider to be most troubling.

We find that the positive relationship between Facebook engagement with a misleading story and belief in the story’s false claims was especially strong for satiric news. We do not have clear evidence about the direction of causality, but either direction is problematic. If social media engagement promotes belief, then high-profile satiric news sites could be inadvertently contributing to a misinformed electorate. If the causal arrow is reversed, and misleading satire tends to attract larger audiences, then satiric news sites have a strong incentive to produce content that people are inclined to believe to generate more revenue. More research into this question is merited.

On a more hopeful note, we find that the “accuracy gap” between Democrats and Republicans for non-satiric misperceptions becomes smaller when we examine belief in claims originating in satire. Republicans are more likely to reject claims originating in satire than other sources, making their performance when assessing satire more similar to Democrats’ performance. This finding is consistent with the observation that Democrats and Republicans are equally capable of understanding humor [34]. If members of both parties understand satire at comparable rates, then they should also be comparably likely to reject literal claims made in satire. This may be less true of falsehoods shared in other types of content.

Finally, we also find that participants were especially good at rejecting claims from satire when its claims had positive social identity implications. Unlike misinformation, individuals were better at rejecting satire that *benefits* their ingroup compared to neutral content. This is a striking contrast to prior work [42]. It would be politically advantageous to interpret satire favoring one’s worldview literally, yet participants did not tend to do so. We speculate this is in part due to the relationship between the satire’s target and its perceived funniness. Messages that benefit one’s worldview are more likely to be seen as funny [43], which makes it more likely that the messages will be written off as “just a joke” [14].

This study demonstrates the importance of examining satire as a distinct source of misperceptions while raising many new questions. For example, do misperceptions based on satire have unique consequences? The unique characteristics of satire suggest that they might. And are existing approaches to preventing or correcting misperceptions more or less effective for satiric content? Given that corrections are more effective when they address the reason for the misperception [44], labeling content as satire should be uniquely effective. Future work should consider how message type may affect our understanding of the formation, prevention, and correction of misperceptions.

### Limitations

There are some important limitations to this study. The analyses conducted here are cross-sectional which prevents us from making claims about the causal ordering of the associations observed. In this way, the work is best understood as complementing experimental studies of individuals’ ability to recognize satire. The study is also limited by its reliance on self-reported media usage which is known to be biased [45]. This means that the relationships with news use observed here may have more to do with biases in self-reports than with real news consumption behaviors. Research using observational data on how people respond to satiric messages would be a valuable addition.

Another potential limitation is that the satire-based statements used to assess participants’ belief were intentionally non-humorous. Our focus was on understanding what American believe about the various claims circulating on social media. Nevertheless, it is possible that we are over-estimating belief by virtue of creating more plausible versions of satiric claims. Still, the influence of individual and message characteristics on belief varied depending on whether the falsehood was based on political satire does suggest that the source on which false claims are based matters. Although the statements that we presented to participants did not include obvious markers of humor, we were still able to detect differences in how participants responded to them.

Our results regarding social media use may also be limited. Our study only used measures of social media use concerning Facebook, Twitter, and Reddit to conceptualize social media use. It is possible that this measurement strategy does not capture the broad range of social media platforms that participants may use. However, prior work has shown that these are widely used platforms [46] and that asking participants about particular specific behaviors they have engaged in is often preferable to more general questions [47]. Future work may endeavor to understand how social media use more broadly interacts with satire to affect beliefs using other measurement strategies.

Lastly, due to our use of non-probability sampling and exclusion of subjects based on missing responses, our results may be specific to the participants in our sample rather than generalizable to our population of interest. To fully understand these phenomena, future work should consider using probability sampling and treating missing data in alternative ways.

## Conclusions

Political satire is a well-known form of humor that can bring levity to otherwise serious (and, in the eyes of many, boring) topics. In doing so, satire can draw in citizens who might otherwise be political uninterested, and it can even help to inform [48]. Unfortunately, as this study illustrates, satire can also mislead. Although most Americans understand that claims made in satiric news articles are false, a non-trivial number express belief in them, often with high confidence.

Further, this study demonstrates that several factors that promote falsehoods have a unique relationship with beliefs based on literal interpretation of satire: some characteristics have a weaker association when messages are satiric while others are stronger. Perhaps most troubling, belief in falsehoods is more strongly correlated with engagement with the source of the claim on Facebook (e.g., liking a satiric news story) when the source is satiric than when it is not. These results illustrate the importance of continued research into the real-world consequences of political satire.

## Supporting information

S1 Appendix Additional stimuli information and robustness checks. (PDF)
