# Supplementary material for: Comparing beliefs in falsehoods based on satiric and non-satiric news
Source: PLoS One. 2023 Jan 19;18(1):e0278639. doi: 10.1371/journal.pone.0278639 (PMC9851529; doi:10.1371/journal.pone.0278639)
Supplement: S1 Appendix — (PDF) [file pone.0278639.s001.pdf]

Supplementary Information File for

# Comparing beliefs in falsehoods based on satiric and non-satiric news

**This PDF file includes:**

Tables S1 to S14
Figures S1 to S4

## Supplementary Tables

**Table S1. False statements wording.**

| Wave | # | Slant | False Statement [a] |
|---|---|---|---|
| 1 | 1 | D | Most middle-class Americans saw their taxes increase in 2018. |
| **1** | **2** | **D** | **Alexandria Ocasio-Cortez has released a plan to provide completely free renewable electricity to every US citizen.** |
| **1** | **3** | **R** | **In her own racial scandal, Elizabeth Warren admitted to wearing paleface at a college costume party.** |
| **1** | **4** | **R** | **To avoid having to answer reporters' questions about her Green New Deal, Rep. Alexandria-Ocasio Cortez pretended that she didn't speak English.** |
| 1 | 5 | R | Apple gave the Mueller investigation unlimited access to Roger Stone's iCloud data, despite refusing to turn over the same kinds of information about known terrorists. |
| 1 | 6 | R | Representative Adam Schiff (D) has been found guilty of conspiring to falsify evidence related to the Trump-Russia investigation. |
| 1 | 7 | N | In a recent survey, 5 million Latin Americans admitted that they plan to enter the US illegally in 2019. |
| 1 | 8 | R | Yale's university newspaper recently published an article urging students to help keep white men out of power by lying about their past behavior. |
| 1 | 9 | D | A UN peacekeeper recently stepped forward with evidence of a sex trafficking operation that has ties to the US State Department. |
| 1 | 10 | R | Rep. Ilhan Omar recently called for the elimination of the Department of Homeland Security. |
| 2 | 1 | N | Under current U.S. law, doctors can legally deny care to an infant born alive during or after an attempted abortion. |
| 2 | 2 | D | Most middle-class Americans saw their taxes increase in 2018. |

| | | | |
|---|---|---|---|
| 2 | 3 | N | At a Black History Month reception last month, President Trump raised his fist in support of the Black Power movement and said it was time for all Americans to join the fight against white nationalism. |
| 2 | 4 | N | A U.S. District court has ruled that abortion opponents have the right to refuse to pay federal taxes on religious grounds because the funds could be used to pay for abortions. |
| 2 | 5 | N | An FEC investigation has concluded that Alexandria Ocasio-Cortez violated campaign finance laws while running for a seat in Congress. |
| 2 | 6 | R | Democrats in California have introduced state legislation intended to protect pedophiles who rape children from having to register as sex offenders. |
| **2** | **7** | **N** | **Most of the money that Bernie Sanders raised immediately after announcing his presidential bid was donated in the Venezuelan currency, leading his campaign to dramatically overstate the size of the contribution.** |
| 2 | 8 | R | Alexandria Ocasio-Cortez claims to live in the Bronx, but evidence shows that she has never lived in that New York City borough. |
| 2 | 9 | N | The U.S. completed construction of hundreds of miles of new border wall on a previously unprotected portion of the US-Mexico border in January 2019. |
| 2 | 10 | D | Leaked emails show that Senate Majority Leader Mitch McConnell used his political position to promote his wife's Kentucky-based business. |
| 3 | 1 | R | Actress Brie Larson, star of Captain Marvel, said that the new film isn't supposed to appeal to white men, and that she doesn't want them to see it. |
| **3** | **2** | **D** | **Tucker Carlson repeatedly criticized a former child bride, who was a guest on his show, for saying that sexual encounters with her adult husband constituted rape.** |
| 3 | 3 | N | In a recent Pew public opinion poll, many men said they were less likely to marry because they were uncomfortable with the idea of their wives working outside the home. |

| | | | |
|---|---|---|---|
| 3 | 4 | D | Environmental activist and Greenpeace ally Patrick Moore recently stepped forward with evidence that human-caused climate change is a hoax. |
| 3 | 5 | N | Nancy Pelosi has advanced legislation that would give illegal immigrants the right to vote in presidential elections |
| 3 | 6 | R | Addressing the Council on American-Islamic Relations, Representative Rashida Tlaib characterized her election as a first step to undermining the U.S. government. |
| 3 | 7 | R | In 2018, the U.S. spent almost twice as much money on welfare for households headed by immigrants as it did supporting families headed by someone born in the U.S. |
| **3** | **8** | **N** | **Senator Kamala Harris has sponsored a bill that would require police officers to get permission from their shift supervisor before drawing a weapon in self-defense.** |
| 3 | 9 | N | Senator Lindsey Graham says that laws promoting the confiscation of numerous types of guns are something that both Democrats and Republicans can get behind. |
| 3 | 10 | R | Muslim immigrants rioted for at least four nights in Grenoble, France after police tried to enter one of the city’s many no-go zones. |
| 4 | 1 | R | There are suspicious circumstances surrounding the apparent suicide of Professor Alan Krueger, a highly regarded economist and top adviser to former Presidents Obama and Clinton. |
| 4 | 2 | N | Senator Dianne Feinstein has called for the confiscation of all guns in an effort to reduce mass shootings. |
| 4 | 3 | R | The Chinese government signed a $1B private equity deal with Hunter Biden’s firm in exchange for then-Vice President Joe Biden’s public support of pro-Chinese trade policies. |
| 4 | 4 | R | Research shows that Google is intentionally manipulating search results in an effort to promote Democratic candidates and liberal politics. |

| | | | |
|---|---|---|---|
| **4** | **5** | **N** | **Most Americans believe that major media companies should apologize for pushing the now-debunked news story of collusion between President Trump and Russia.** |
| 4 | 6 | R | While serving as Sec. of State, Hillary Clinton colluded with Russia, selling 20% of the U.S. uranium supply to that country in exchange for donations to the Clinton Foundation. |
| 4 | 7 | R | Two police officers in Britain recently questioned a man in his home because he expressed support for Brexit in an online discussion forum. |
| 4 | 8 | D | Environmental activist and Greenpeace ally Patrick Moore recently stepped forward with evidence that human-caused climate change is a hoax. |
| 4 | 9 | R | Both Barack and Michelle Obama's admission to Harvard University was part of an admissions scandal dating back to the late 1980s. |
| **4** | **10** | **R** | **Elizabeth Warren has proposed amending the Constitution to prohibit the election of Republicans, eliciting praise from several other prominent Democrats.** |
| **5** | **1** | **N** | **Protesters in Buffalo, NY, claimed a major victory after blocking customers and employees from entering a local Chick-fil-A restaurant for one day.** |
| **5** | **2** | **D** | **White House Press Secretary Sarah Huckabee-Sanders told reporters that releasing the Mueller report would be a threat to public safety because so many Americans would be disturbed by evidence of the president's innocence.** |
| **5** | **3** | **R** | **The White House announced that it was appointing Joe Biden to serve as the head of the Transportation Security Agency (TSA), citing his comfort working closely with people.** |
| 5 | 4 | R | While Vice President, Joe Biden directed $1.8B in US aid to Ukraine in exchange for payments to his son from a Ukrainian energy company totaling at least $3.1M. |
| 5 | 5 | N | A Canadian man was fined $55,000 for describing a transgender female political candidate as male. |

| | | | |
|---|---|---|---|
| **5** | **6** | **D** | **Televangelist Pat Robertson claims to have had a vision of Jesus wearing camo and carrying an AK-47, signaling that the Second Coming is near.** |
| 5 | 7 | D | In early 2019, a Kansas court sentenced a 23-year-old black man to 12 years in prison for kissing a 17-year-old white girl at a bar. |
| 5 | 8 | R | The Russian government invested $35M in a US energy firm linked to Clinton campaign manager John Podesta in exchange for his consistent support of Russian interests. |
| 5 | 9 | N | With a job approval rating of 53%, President Trump is now viewed more favorably than President Obama was during his second year in office. |
| 5 | 10 | R | Current CO2 levels of 410 parts per million were previously seen on Earth three million years ago, which means that humans are not responsible for climate change. |
| **6** | **1** | **R** | **Bernie Sanders said that American ISIS members should be allowed to vote in U.S. elections.** |
| **6** | **2** | **N** | **Queen Elizabeth said that she will not meet with President Trump during his state visit to the UK.** |
| **6** | **3** | **N** | **CNN news anchor Anderson Cooper said his belief that Trump colluded with Russia is unshakable; it will not change regardless of statements or evidence to the contrary.** |
| **6** | **4** | **D** | **Speaking at a gathering of wealthy donors, Pope Francis promised that money raised to help rebuild Notre Dame Cathedral will not be used to help the poor.** |
| 6 | 5 | R | British protestors are stockpiling human urine, and are planning to douse President Trump with it during his state visit to the UK in June. |
| 6 | 6 | N | The Center for Disease Control (CDC) says that recent measles outbreaks in the US are the product of international travel, and have little to do with falling vaccination rates. |

| | | | |
|---|---|---|---|
| 6 | 7 | N | Abortion rates are falling dramatically in response to the release of “Unplanned,” a movie about a Planned Parenthood clinic manager who became a pro-life activist. |
| 6 | 8 | R | Over its lifetime, an electric car driven in the U.S. will release more carbon into the atmosphere than a diesel car after accounting for battery production. |
| 6 | 9 | N | Every prediction about humans’ environmental impact made since 1970 has been wrong. |
| 6 | 10 | R | An investigation by the Inspector General concluded that the Justice Department and FBI abused the Foreign Intelligence Surveillance Act (FISA) under President Obama’s direction. |
| 7 | 1 | R | Special counsel Robert Mueller is furious at Nancy Pelosi for lying about Attorney General William Barr and wants to testify before Congress to correct the record. |
| 7 | 2 | R | Facebook has issued a statement defending its users’ right to discuss assassinating President Trump on the platform, saying that such speech does not violate its community guidelines. |
| 7 | 3 | N | Rep. Jerry Nadler shared confidential documents about President Trump that he obtained by subpoena with his son’s law firm, which is currently suing the President. |
| 7 | 4 | R | Hillary Clinton wants China to get President Trump’s tax returns by hacking the IRS, and she says the country should share the stolen documents with the American news media. |
| 7 | 5 | R | Nellie Ohr, wife of Justice Department official Bruce Ohr, has been convicted of lying to Congress when giving testimony about her work for Fusion GPS. |
| **7** | **6** | **R** | **Representative Ilhan Omar said that being Jewish is an inherently hostile act, especially among those living in Israel.** |
| **7** | **7** | **N** | **A SWAT team was sent to arrest a man after Facebook reported him for holding extremely conservative views.** |

| | | | |
|---|---|---|---|
| 7 | 8 | N | Speaker of the House Nancy Pelosi said that Democrats should be prepared to challenge the validity of the 2020 election if Trump wins a second term. |
| 7 | 9 | N | None of the 21 Democrats running for president defended Israel or criticized Hamas during the latest round of rocket attacks on the Gaza strip. |
| 7 | 10 | R | Former Vice President Joe Biden repeatedly referred to British Prime Minister Theresa May as Margaret Thatcher, a former PM who left office in 1990, and appeared to be unaware of his mistake. |
| **8** | **1** | **R** | **Alabama State Senator Katie Shaw proposed an amendment to the state's new abortion bill requiring that unmarried men get vasectomies that could only be reversed when they marry.** |
| **8** | **2** | **R** | **Bernie Sanders has criticized billionaire Robert Smith's promise to pay off Morehouse College graduates' student debt, arguing that the "federal government could put [the money] to better use."** |
| **8** | **3** | **D** | **Following the passage of Alabama's new restrictive abortion bill, a 12-year-old victim of sexual abuse said during an interview that she doesn't think she can be a mom on top of her already hectic life.** |
| 8 | 4 | D | Georgia's new abortion bill requires an investigation of any woman who miscarries in order to identify whether the miscarriage was intentionally induced. |
| 8 | 5 | D | Alabama State Rep. Barry Hovis is vigorously defending his assertion that rape can be "consensual." |
| **8** | **6** | **D** | **National Security Advisor John Bolton said that an attack on two Saudi Arabian oil tankers in the Gulf of Oman is "an attack on all Americans."** |
| **8** | **7** | **R** | **Facebook has admitted holding a party to celebrate the suspension of conservative activist Candace Owen's account.** |
| **8** | **8** | **D** | **A pro-life man told his coworkers that he identifies as a woman in order to get them to listen to his opinions on abortion.** |

| | | | |
|---|---|---|---|
| 8 | 9 | N | A Christian student failed her world history course because she refused to recite an Islamic prayer for an assignment. |
| 8 | 10 | D | Model Emily Ratajkowski posted a message on Twitter suggesting that allowing black women to have abortions is a good way to reduce crime and promote public safety. |
| 9 | 1 | N | Several recent public opinion polls show President Trump's job approval rating to be about 48%, the highest it has been in two years. |
| **9** | **2** | **N** | **Bob Iger, CEO of Disney, said that the company will move its Georgia-based filming operations back to Hollywood in order to avoid filming among "depraved, immoral people."** |
| **9** | **3** | **D** | **President Donald Trump was rushed off stage at a rally after Secret Service agents spotted a man in the audience holding a picture of the late Senator John McCain.** |
| 9 | 4 | R | The Chinese government signed a $1B private equity deal with Hunter Biden's firm in exchange for then-Vice President Joe Biden's public support of pro-Chinese trade policies. |
| **9** | **5** | **R** | **The U.S. Navy has confirmed that vessels controlled by Iran's Revolutionary Guard fired on a pair of U.S. warships in the Gulf of Tonkin, off the coast of Vietnam.** |
| **9** | **6** | **R** | **In celebration of LGBT+ Pride month, Microsoft has made all Xbox gaming account avatars transgender for the month of June.** |
| **9** | **7** | **D** | **Senator Mitch McConnell says he is embarrassed that his wife, U.S. Secretary of Transportation Elaine Chao, is better at getting bribes and other illicit payments than he is.** |
| 9 | 8 | R | Maya Rockeymoore Cummings, wife of Maryland Democratic Representative Elijah Cummings, has been found guilty of funneling millions of dollars in bribes through her non-profit organization. |
| **9** | **9** | **D** | **President Donald Trump has ordered the U.S. Navy to sink the USS John S. McCain, which would make this the first active Navy vessel to be scuttled since World War II.** |

| | | | |
|---|---|---|---|
| 9 | 10 | R | Special Prosecutor Robert Mueller falsified a transcript of a voicemail from President Trump’s attorney, John Dowd, in an effort to frame the President for a crime he did not commit. |
| **10** | **1** | **D** | **Vice President Mike Pence has acknowledged that he routinely visits a gay conversion therapist to help ensure that he never becomes attracted to men.** |
| **10** | **2** | **R** | **In a campaign speech, Joe Biden attempted to praise the Democratic Party, saying that “Republicans may have standards, but we have double standards.** |
| **10** | **3** | **R** | **In a video to her constituents Monday evening, Representative Alexandria Ocasio-Cortez compared mandatory math classes in public schools to "concentration camps."** |
| 10 | 4 | R | In late 2016, a small group of State Department employees loyal to President Obama began planning a coup attempt against President Trump. |
| **10** | **5** | **N** | **Cory Booker is one of several prominent Democrats to describe the Second Amendment as a dangerous “loophole” that allows people to buy guns.** |
| **10** | **6** | **D** | **Following a recommendation that she be fired for repeatedly violating the Hatch Act, White House counselor Kellyanne Conway told reporters that she intends to keep a low profile until, “the laws governing this country have no repercussions.”** |
| **10** | **7** | **N** | **Democratic presidential candidate Elizabeth Warren has promised to cure smallpox as one of her first actions upon taking office.** |
| 10 | 8 | R | Democratic presidential candidate Bernie Sanders’s “Economic Bill of Rights” plagiarizes parts of Russian dictator Joseph Stalin’s Soviet Constitution. |
| **10** | **9** | **D** | **At a recent press conference, John Bolton falsely claimed that the U.S. government has never lied about ships being attacked in order to justify getting involved in a foreign conflict.** |

| | | | |
|---|---|---|---|
| 10 | 10 | D | Most evidence suggests that attacks on two oil tankers in the Gulf of Oman, which Secretary of State Pompeo blamed on Iranian forces, were staged by the U.S. |
| 11 | 1 | R | In a staged photo, Rep. Alexandria Ocasio-Cortez (D) cried while looking through a fence toward what she pretended was a detention facility housing children in Texas. |
| **11** | **2** | **R** | **During the first round of Democratic debates, Beto O'Rourke chugged a bottle of Cholula Hot Sauce in an effort to appeal to Hispanic voters.** |
| 11 | 3 | N | In a recent Supreme Court decision, Justice Clarence Thomas said that abortion is most accurately described as the "dismemberment of a living child." |
| **11** | **4** | **N** | **House Democrats voted in support of an emergency spending bill promoting the use of photography to document how upset they are by the treatment of immigrants entering the U.S. illegally.** |
| **11** | **5** | **R** | **Hillary Clinton has proposed reparations—payments intended to make amends for a past injustice—to anyone who lost a presidential election to President Trump.** |
| 11 | 6 | N | The U.S. government spends approximately $18.5 billion a year on programs designed to provide health care for immigrants who entered the country illegally. |
| **11** | **7** | **N** | **A female server at an upscale Chicago restaurant accused of spitting on Eric Trump has denied the charge, saying simply that Trump is "not my type."** |
| 11 | 8 | R | During the Democratic presidential debates, California Sen. Kamala Harris (D) lied about being bused to school in Berkeley, CA as a child. |
| 11 | 9 | R | Candidates participating in the Democratic primary debates were uniformly in favor of taking health insurance away from native-born Americans and giving it to undocumented immigrants instead. |

| | | | |
|---|---|---|---|
| 11 | 10 | N | Millions of Americans are calling for Rep. Ocasio-Cortez (D) to resign after she lied about the treatment of migrants in Customs and Border Patrol detention centers. |
| **12** | **1** | **N** | **The Trump reelection campaign is planning a series of ads featuring unedited footage of speeches and press conferences by “crazy” liberal Democrats such as Alexandria Ocasio-Cortez, Ilhan Omar, and Rashida Tlaib.** |
| 12 | 2 | R | Reps. Alexandria Ocasio-Cortez (D) and Ilhan Omar (D) have praised antifa groups for promoting a violent attack on an ICE detention center in Seattle. |
| **12** | **3** | **R** | **The US women's soccer team filed a lawsuit to overturn the “unjust, unfair, unconstitutional law of supply and demand,” which states that prices tend to be higher for products that are more popular.** |
| 12 | 4 | R | U.S. Women’s National Team soccer star and World Cup Champion Megan Rapinoe has frequently boasted about convincing people to be unpatriotic and anti-American. |
| **12** | **5** | **R** | **During an open session of Congress, Rep. Alexandria Ocasio-Cortez exclaimed, “Wow! We pay soldiers way too much!”** |
| 12 | 6 | R | Soccer player Megan Rapinoe said that science has proven that gay people are better athletes than straight people. |
| **12** | **7** | **D** | **In selecting its new ambassador to the U.S., U.K. officials have admitted that they prioritized finding someone who would be willing to “pretend that Donald Trump isn’t a moron.”** |
| 12 | 8 | N | There is near-consensus among economists that, over the long-term, deporting undocumented immigrants is more economically beneficial to the U.S. than providing a path to citizenship. |
| **12** | **9** | **D** | **Speaking to a group of migrant children held at an ICE detention center, Vice President Pence assured the young detainees that they will have safe and sanitary conditions in heaven.** |

| | | | |
|---|---|---|---|
| 12 | 10 | D | **Polls show that President Trump's supporters are worried he won't follow through on his threat to send four Democratic minority congresswomen "back to the countries they came from."** |

Statements in bold originate from satire.

[a] A political claim is false if it is inconsistent with the conclusions of people holding relevant expertise, including journalists, scientists, and eyewitnesses. Slant refers to how the statement was classified by crowdworkers on Amazon Mechanical Turk.

**Table S2. Sources of stories on which statements were based.**

| **Satire** | **Non-satire** |
|---|---|
| alternativelyfacts.com | afa.net |
| babylonbee.com | africanglobe.net |
| bizstandardnews.com | breitbart.com |
| bluenewsnetwork.com | christianheadlines.com |
| breakingburgh.com | collective-evolution.com |
| chaser.com.au | dailywire.com |
| duffelblog.com | disclose.tv |
| genesiustimes.com | foxnews.com |
| huzlers.com | harpersbazaar.com |
| newsthump.com | ilovemyfreedom.org |
| newyorker.com | infowars.com |
| tatersgonnatate.com | madworldnews.com |
| thebeaverton.com | nbcnews.com |
| theonion.com | newspunch.com |
| | newstarget.com |
| | newswars.com |
| | nypost.com |
| | politico.com |
| | rt.com |
| | sandrarose.com |
| | sputniknews.com |
| | teaparty.org |
| | theamericanmirror.com |
| | theconservativeopinion.com |
| | theepochtimes.com |
| | thefederalistpapers.org |
| | thegatewaypundit.com |
| | thehill.com |

| | |
|---|---|
| | thewashingtonpundit.com |
| | washingtonpost.com |
| | wattsupwiththat.com |
| | wnd.com |
| | wokesloth.com |
| | zerohedge.com |

**Table S3. Mixed effects regression model of belief in falsehoods with interaction between story type and age.**

| | **Coefficient** | **Std. error** | ***P* value** | **95% CI** |
|---|---|---|---|---|
| **Satire** | -0.012 | 0.036 | 0.745 | -0.082, 0.059 |
| **Education** | -0.025 | 0.010 | 0.016 | -0.046 -0.005 |
| **Political interest** | -0.076 | 0.018 | <0.001 | -0.112, -0.040 |
| **Sex (male = 1)** | -0.056 | 0.029 | 0.059 | -0.113, 0.002 |
| **Age** | -0.001 | 0.001 | 0.436 | -0.003, 0.001 |
| **Age x Satire** | -0.001 | 0.001 | 0.115 | -0.002, 0.000 |
| **Party ID (Democrat = 1)** | -0.239 | 0.030 | <0.001 | -0.298, -0.179 |
| **Ingroup-beneficial statement** | 0.103 | 0.012 | <0.001 | 0.080, 0.126 |
| **Ingroup-harmful statement** | -0.236 | 0.011 | <0.001 | -0.258, -0.214 |
| **Faith in Intuition for Facts** | 0.100 | 0.024 | <0.001 | 0.054, 0.146 |
| **Need for Evidence** | -0.019 | 0.026 | 0.463 | -0.069, 0.031 |
| **Truth is Political** | 0.085 | 0.020 | <0.001 | 0.046, 0.125 |
| **Conspiracy mentality** | 0.148 | 0.024 | <0.001 | 0.101, 0.195 |
| **Average online news use** | 0.004 | 0.005 | 0.389 | -0.006, 0.015 |
| **Average offline news use** | 0.027 | 0.012 | 0.022 | 0.004, 0.049 |
| **Average social media use** | 0.024 | 0.014 | 0.100 | -0.005, 0.052 |
| **Facebook engagement (log)** | 0.035 | 0.004 | <0.001 | 0.028, 0.042 |
| **Intercept** | | | <0.001 | 1.091, 1.778 |
| **Number of observations** | 50926.000 | | | |
| **Number of clusters** | 480.000 | | | |
| $\chi^2$ | 1167.862 | | | |
| **Model test *p* value** | <0.001 | | | |
| **AIC** | 110428.2 | | | |
| **BIC** | 110613.8 | | | |

**Table S4. Mixed effects regression model of belief in falsehoods with interaction between story type and whether statement benefits political ingroup**

| | **Coefficient** | **Std. error** | ***P* value** | **95% CI** |
|---|---|---|---|---|
| **Satire** | -0.001 | 0.010 | 0.909 | -0.020, 0.018 |
| **Education** | -0.025 | 0.010 | 0.017 | -0.045, -0.005 |
| **Political interest** | -0.076 | 0.018 | <0.001 | -0.112, -0.040 |
| **Sex (male = 1)** | -0.056 | 0.029 | 0.059 | -0.113, 0.002 |
| **Age** | -0.001 | 0.001 | 0.237 | -0.003, 0.001 |
| **Party ID (Democrat = 1)** | -0.219 | 0.030 | <0.001 | -0.278, -0.161 |
| **Ingroup-beneficial statement** | 0.187 | 0.015 | <0.001 | 0.158, 0.215 |
| **Ingroup-beneficial statement x Satire** | -0.196 | 0.015 | <0.001 | -0.225, -0.167 |
| **Ingroup-harmful statement** | -0.245 | 0.012 | <0.001 | -0.267, -0.222 |
| **Faith in Intuition for Facts** | 0.100 | 0.024 | <0.001 | 0.054, 0.146 |
| **Need for Evidence** | -0.019 | 0.026 | 0.462 | -0.069, 0.031 |
| **Truth is Political** | 0.086 | 0.020 | <0.001 | 0.046, 0.125 |
| **Conspiracy mentality** | 0.148 | 0.024 | <0.001 | 0.101, 0.194 |
| **Average online news use** | 0.004 | 0.005 | 0.420 | -0.006, 0.014 |
| **Average offline news use** | 0.026 | 0.012 | 0.024 | 0.003, 0.049 |
| **Average social media use** | 0.024 | 0.014 | 0.099 | -0.004, 0.052 |
| **Facebook engagement (log)** | 0.035 | 0.004 | <0.001 | 0.028, 0.042 |
| **Intercept** | | | <0.001 | 1.087, 1.769 |
| **Number of observations** | 50926.000 | | | |
| **Number of clusters** | 480.000 | | | |
| $\chi^2$ | 1194.413 | | | |
| **Model test *p* value** | <0.001 | | | |
| **AIC** | 110222.6 | | | |
| **BIC** | 110408.2 | | | |

**Table S5. Mixed effects regression model of belief in falsehoods with interaction between story type and whether statement harms ingroup.**

| | Coefficient | Std. error | *P* value | 95% CI |
|---|---|---|---|---|
| **Satire** | -0.071 | 0.012 | <0.001 | -0.094, -0.047 |
| **Education** | -0.025 | 0.010 | 0.016 | -0.046, -0.005 |
| **Political interest** | -0.076 | 0.018 | <0.001 | -0.112, -0.040 |
| **Sex (male = 1)** | -0.056 | 0.029 | 0.059 | -0.113, 0.002 |
| **Age** | -0.001 | 0.001 | 0.234 | -0.003, 0.001 |
| **Party ID (Democrat = 1)** | -0.238 | 0.030 | <0.001 | -0.297, -0.179 |
| **Ingroup-beneficial statement** | 0.104 | 0.012 | <0.001 | 0.081, 0.126 |
| **Ingroup-harmful statement** | -0.239 | 0.013 | <0.001 | -0.265, -0.213 |
| **Satire x ingroup-harmful statement** | 0.006 | 0.013 | 0.640 | -0.019, 0.031 |
| **Faith in Intuition for Facts** | 0.100 | 0.024 | <0.001 | 0.054, 0.146 |
| **Need for Evidence** | -0.019 | 0.026 | 0.464 | -0.069, 0.031 |
| **Truth is Political** | 0.086 | 0.020 | <0.001 | 0.046, 0.125 |
| **Conspiracy mentality** | 0.148 | 0.024 | <0.001 | 0.101, 0.195 |
| **Average online news use** | 0.004 | 0.005 | 0.400 | -0.006, 0.015 |
| **Average offline news use** | 0.027 | 0.012 | 0.022 | 0.004, 0.049 |
| **Average social media use** | 0.024 | 0.014 | 0.100 | -0.005, 0.052 |
| **Facebook engagement (log)** | 0.035 | 0.003 | <0.001 | 0.028, 0.042 |
| **Intercept** | | | <0.001 | 1.118, 1.799 |
| **Number of observations** | 50926.000 | | | |
| **Number of clusters** | 480.000 | | | |
| $\chi^2$ | 1170.458 | | | |
| **Model test *p* value** | <0.001 | | | |
| **AIC** | 110434 | | | |
| **BIC** | 110619.6 | | | |

**Table S6. Mixed effects regression model of belief in falsehoods with interaction between story type and party identification.**

| | Coefficient | Std. error | *P* value | 95% CI |
|---|---|---|---|---|
| **Satire** | -0.187 | 0.015 | <0.001 | -0.216, -0.157 |
| **Education** | -0.025 | 0.010 | 0.016 | -0.046, -0.005 |
| **Political interest** | -0.076 | 0.018 | <0.001 | -0.113, -0.040 |
| **Sex (male = 1)** | -0.056 | 0.029 | 0.059 | -0.113, 0.002 |
| **Age** | -0.001 | 0.001 | 0.227 | -0.003, 0.001 |
| **Party ID (Democrat = 1)** | -0.332 | 0.031 | <0.001 | -0.393, -0.272 |
| **Party ID x Satire** | 0.218 | 0.017 | <0.001 | 0.184, 0.252 |
| **Ingroup-beneficial statement** | 0.090 | 0.011 | <0.001 | 0.068, 0.113 |
| **Ingroup-harmful statement** | -0.224 | 0.011 | <0.001 | -0.246, -0.203 |
| **Faith in Intuition for Facts** | 0.100 | 0.024 | <0.001 | 0.054, 0.146 |
| **Need for Evidence** | -0.018 | 0.026 | 0.470 | -0.068, 0.032 |
| **Truth is Political** | 0.085 | 0.020 | <0.001 | 0.046, 0.125 |
| **Conspiracy mentality** | 0.148 | 0.024 | <0.001 | 0.101, 0.195 |
| **Average online news use** | 0.005 | 0.005 | 0.346 | -0.005, 0.015 |
| **Average offline news use** | 0.027 | 0.012 | 0.022 | 0.004, 0.049 |
| **Average social media use** | 0.022 | 0.014 | 0.119 | -0.006, 0.050 |
| **Facebook engagement (log)** | 0.035 | 0.004 | <0.001 | 0.028, 0.042 |
| **Intercept** | | | <0.001 | 1.167, 1.849 |
| **Number of observations** | 50926.000 | | | |
| **Number of clusters** | 480.000 | | | |
| $\chi^2$ | 1197.418 | | | |
| **Model test *p* value** | <0.001 | | | |
| **AIC** | 110159.1 | | | |
| **BIC** | 110344.7 | | | |

**Table S7. Mixed effects regression model of belief in falsehoods with interaction between story type and Faith in Intution for Facts.**

| | Coefficient | Std. error | *P* value | 95% CI |
|---|---|---|---|---|
| **Satire** | 0.184 | 0.054 | 0.001 | 0.077, 0.290 |
| **Education** | -0.025 | 0.010 | 0.017 | -0.045, -0.005 |
| **Political interest** | -0.076 | 0.018 | <0.001 | -0.112, -0.040 |
| **Sex (male = 1)** | -0.055 | 0.029 | 0.060 | -0.113, 0.002 |
| **Age** | -0.001 | 0.001 | 0.237 | -0.003, 0.001 |
| **Party ID (Democrat = 1)** | -0.239 | 0.030 | <0.001 | -0.298, -0.180 |
| **Ingroup-beneficial statement** | 0.102 | 0.012 | <0.001 | 0.079, 0.125 |
| **Ingroup-harmful statement** | -0.235 | 0.011 | <0.001 | -0.257, -0.213 |
| **Faith in Intuition for Facts** | 0.130 | 0.024 | <0.001 | 0.083, 0.178 |
| **Faith in Intuition for Facts x Satire** | -0.074 | 0.016 | <0.001 | -0.106, -0.043 |
| **Need for Evidence** | -0.019 | 0.026 | 0.461 | -0.069, 0.031 |
| **Truth is Political** | 0.086 | 0.020 | <0.001 | 0.046, 0.125 |
| **Conspiracy mentality** | 0.147 | 0.024 | <0.001 | 0.100, 0.194 |
| **Average online news use** | 0.004 | 0.005 | 0.413 | -0.006, 0.014 |
| **Average offline news use** | 0.026 | 0.012 | 0.025 | 0.003, 0.049 |
| **Average social media use** | 0.024 | 0.014 | 0.100 | -0.004, 0.052 |
| **Facebook engagement (log)** | 0.035 | 0.004 | <0.001 | 0.028, 0.042 |
| **Intercept** | | | <0.001 | 1.015, 1.698 |
| **Number of observations** | 50926.000 | | | |
| **Number of clusters** | 480.000 | | | |
| $\chi^2$ | 1188.953 | | | |
| **Model test *p* value** | <0.001 | | | |
| **AIC** | 110376.5 | | | |
| **BIC** | 110562.1 | | | |

**Table S8. Mixed effects regression model of belief in falsehoods with interaction between story type and Need for Evidence.**

| | Coefficient | Std. error | *P* value | 95% CI |
|---|---|---|---|---|
| **Satire** | -0.199 | 0.067 | 0.003 | -0.331, -0.067 |
| **Education** | -0.025 | 0.010 | 0.016 | -0.046, -0.005 |
| **Political interest** | -0.076 | 0.018 | <0.001 | -0.112, -0.040 |
| **Sex (male = 1)** | -0.056 | 0.029 | 0.059 | -0.113, 0.002 |
| **Age** | -0.001 | 0.001 | 0.234 | -0.003, 0.001 |
| **Party ID (Democrat = 1)** | -0.239 | 0.030 | <0.001 | -0.298, -0.179 |
| **Ingroup-beneficial statement** | 0.103 | 0.012 | <0.001 | 0.080, 0.126 |
| **Ingroup-harmful statement** | -0.236 | 0.011 | <0.001 | -0.258, -0.214 |
| **Faith in Intuition for Facts** | 0.100 | 0.024 | <0.001 | 0.054, 0.146 |
| **Need for Evidence** | -0.032 | 0.027 | 0.236 | -0.085, 0.021 |
| **Need for Evidence x Satire** | 0.033 | 0.017 | 0.055 | -0.001, 0.066 |
| **Truth is Political** | 0.086 | 0.020 | <0.001 | 0.046, 0.125 |
| **Conspiracy mentality** | 0.148 | 0.024 | 0.000 | 0.101, 0.195 |
| **Average online news use** | 0.004 | 0.005 | 0.398 | -0.006, 0.015 |
| **Average offline news use** | 0.027 | 0.012 | 0.022 | 0.004, 0.049 |
| **Average social media use** | 0.023 | 0.014 | 0.101 | -0.005, 0.052 |
| **Facebook engagement (log)** | 0.035 | 0.004 | <0.001 | 0.028, 0.042 |
| **Intercept** | | | <0.001 | 1.163, 1.858 |
| **Number of observations** | 50926.000 | | | |
| **Number of clusters** | 480.000 | | | |
| $\chi^2$ | 1163.842 | | | |
| **Model test *p* value** | <0.001 | | | |
| **AIC** | 110424.4, | | | |
| **BIC** | 110610 | | | |

**Table S9. Mixed effects regression model of belief in falsehoods with interaction between story type and Truth is Political.**

| | Coefficient | Std. error | *P* value | 95% CI |
|---|---|---|---|---|
| **Satire** | 0.081 | 0.035 | 0.022 | 0.011, 0.151 |
| **Education** | -0.025 | 0.010 | 0.016 | -0.045, -0.005 |
| **Political interest** | -0.076 | 0.018 | <0.001 | -0.112, -0.040 |
| **Sex (male = 1)** | -0.056 | 0.029 | 0.059 | -0.113, 0.002 |
| **Age** | -0.001 | 0.001 | 0.232 | -0.003, 0.001 |
| **Party ID (Democrat = 1)** | -0.239 | 0.030 | <0.001 | -0.298, -0.180 |
| **Ingroup-beneficial statement** | 0.102 | 0.012 | <0.001 | 0.079, 0.124 |
| **Ingroup-harmful statement** | -0.235 | 0.011 | <0.001 | -0.257, -0.213 |
| **Faith in Intuition for Facts** | 0.100 | 0.024 | <0.001 | 0.054, 0.146 |
| **Need for Evidence** | -0.019 | 0.026 | 0.465 | -0.069, 0.031 |
| **Truth is Political** | 0.108 | 0.021 | <0.001 | 0.067, 0.149 |
| **Truth is Political x Satire** | -0.055 | 0.013 | <0.001 | -0.080, -0.030 |
| **Conspiracy mentality** | 0.147 | 0.024 | <0.001 | 0.100, 0.194 |
| **Average online news use** | 0.004 | 0.005 | 0.402 | -0.006, 0.015 |
| **Average offline news use** | 0.027 | 0.012 | 0.020 | 0.004, 0.050 |
| **Average social media use** | 0.024 | 0.014 | 0.097 | -0.004, 0.052 |
| **Facebook engagement (log)** | 0.035 | 0.004 | <0.001 | 0.028, 0.042 |
| **Intercept** | | | <0.001 | 1.057, 1.736 |
| **Number of observations** | 50926.000 | | | |
| **Number of clusters** | 480.000 | | | |
| **$\chi^2$** | 1202.428 | | | |
| **Model test *p* value** | <0.001 | | | |
| **AIC** | 110379 | | | |
| **BIC** | 110564.6 | | | |

**Table S10. Mixed effects regression model of belief in falsehoods with interaction between story type and Conspiracy Mentality.**

| | Coefficient | Std. error | *P* value | 95% CI |
|---|---|---|---|---|
| **Satire** | 0.156 | 0.058 | 0.008 | 0.041, 0.270 |
| **Education** | -0.025 | 0.010 | 0.017 | -0.045, -0.005 |
| **Political interest** | -0.076 | 0.018 | <0.001 | -0.112, -0.040 |
| **Sex (male = 1)** | -0.055 | 0.029 | 0.059 | -0.113, 0.002 |
| **Age** | -0.001 | 0.001 | 0.233 | -0.003, 0.001 |
| **Party ID (Democrat = 1)** | -0.239 | 0.030 | <0.001 | -0.298, -0.180 |
| **Ingroup-beneficial statement** | 0.103 | 0.012 | <0.001 | 0.080, 0.125 |
| **Ingroup-harmful statement** | -0.236 | 0.011 | <0.001 | -0.258, -0.214 |
| **Faith in Intuition for Facts** | 0.100 | 0.024 | <0.001 | 0.054, 0.146 |
| **Need for Evidence** | -0.019 | 0.026 | 0.464 | -0.069, 0.031 |
| **Truth is Political** | 0.086 | 0.020 | <0.001 | 0.046, 0.125 |
| **Conspiracy mentality** | 0.171 | 0.025 | <0.001 | 0.123, 0.220 |
| **Conspiracy mentality x Satire** | -0.059 | 0.016 | <0.001 | -0.090, -0.029 |
| **Average online news use** | 0.004 | 0.005 | 0.413 | -0.006, 0.015 |
| **Average offline news use** | 0.027 | 0.012 | 0.022 | 0.004, 0.049 |
| **Average social media use** | 0.024 | 0.014 | 0.100 | -0.005, 0.052 |
| **Facebook engagement (log)** | 0.035 | 0.004 | <0.001 | 0.028, 0.042 |
| **Intercept** | | | <0.001 | 1.024, 1.713 |
| **Number of observations** | 50926.000 | | | |
| **Number of clusters** | 480.000 | | | |
| $\chi^2$ | 1183.164 | | | |
| **Model test *p* value** | <0.001 | | | |
| **AIC** | 110397.4 | | | |
| **BIC** | 110583 | | | |

**Table S11. Mixed effects regression model of belief in falsehoods with interaction between story type and average online news use.**

| | **Coefficient** | **Std. error** | ***P* value** | **95% CI** |
|---|---|---|---|---|
| **Satire** | -0.027 | 0.019 | 0.140 | -0.064, 0.009 |
| **Education** | -0.025 | 0.010 | 0.016 | -0.046, -0.005 |
| **Political interest** | -0.076 | 0.018 | <0.001 | -0.112, -0.040 |
| **Sex (male = 1)** | -0.056 | 0.029 | 0.059 | -0.113, 0.002 |
| **Age** | -0.001 | 0.001 | 0.235 | -0.003, 0.001 |
| **Party ID (Democrat = 1)** | -0.239 | 0.030 | <0.001 | -0.298, -0.180 |
| **Ingroup-beneficial statement** | 0.103 | 0.012 | <0.001 | 0.080, 0.126 |
| **Ingroup-harmful statement** | -0.236 | 0.011 | <0.001 | -0.258, -0.214 |
| **Faith in Intuition for Facts** | 0.100 | 0.024 | <0.001 | 0.054, 0.146 |
| **Need for Evidence** | -0.019 | 0.026 | 0.462 | -0.069, 0.031 |
| **Truth is Political** | 0.086 | 0.020 | <0.001 | 0.046, 0.125 |
| **Conspiracy mentality** | 0.148 | 0.024 | <0.001 | 0.101, 0.195 |
| **Average online news use** | 0.010 | 0.006 | 0.082 | -0.001, 0.021 |
| **Average online news use x Satire** | -0.014 | 0.006 | 0.024 | -0.025, -0.002 |
| **Average offline news use** | 0.027 | 0.012 | 0.021 | 0.004, 0.049 |
| **Average social media use** | 0.024 | 0.014 | 0.099 | -0.004, 0.052 |
| **Facebook engagement (log)** | 0.035 | 0.004 | <0.001 | 0.028, 0.042 |
| **Intercept** | | | <0.001 | 1.100, 1.781 |
| **Number of observations** | 50926.000 | | | |
| **Number of clusters** | 480.000 | | | |
| $\chi^2$ | 1167.874 | | | |
| **Model test *p* value** | <0.001 | | | |
| **AIC** | 110423.2 | | | |
| **BIC** | 110608.8 | | | |

**Table S12. Mixed effects regression model of belief in falsehoods with interaction between story type and average offline news use.**

| | Coefficient | Std. error | *P* value | 95% CI |
|---|---|---|---|---|
| **Satire** | -0.054 | 0.026 | 0.036 | -0.105, -0.004 |
| **Education** | -0.025 | 0.010 | 0.016 | -0.046, -0.005 |
| **Political interest** | -0.076 | 0.018 | <0.001 | -0.112, -0.040 |
| **Sex (male = 1)** | -0.056 | 0.029 | 0.059 | -0.113, 0.002 |
| **Age** | -0.001 | 0.001 | 0.235 | -0.003, 0.001 |
| **Party ID (Democrat = 1)** | -0.239 | 0.030 | <0.001 | -0.298, -0.179 |
| **Ingroup-beneficial statement** | 0.103 | 0.012 | <0.001 | 0.080, 0.126 |
| **Ingroup-harmful statement** | -0.236 | 0.011 | <0.001 | -0.258, -0.214 |
| **Faith in Intuition for Facts** | 0.100 | 0.024 | <0.001 | 0.054, 0.146 |
| **Need for Evidence** | -0.019 | 0.026 | 0.463 | -0.069, 0.031 |
| **Truth is Political** | 0.086 | 0.020 | <0.001 | 0.046, 0.125 |
| **Conspiracy mentality** | 0.148 | 0.024 | <0.001 | 0.101, 0.195 |
| **Average online news use** | 0.004 | 0.005 | 0.396 | -0.006, 0.015 |
| **Average offline news use** | 0.029 | 0.012 | 0.017 | 0.005, 0.053 |
| **Average offline news use x Satire** | -0.006 | 0.010 | 0.563 | -0.026, 0.014 |
| **Average social media use** | 0.024 | 0.014 | 0.100 | -0.004, 0.052 |
| **Facebook engagement (log)** | 0.035 | 0.004 | <0.001 | 0.028, 0.042 |
| **Intercept** | | | <0.001 | 1.109, 1.794 |
| **Number of observations** | 50926.000 | | | |
| **Number of clusters** | 480.000 | | | |
| $\chi^2$ | 1171.366 | | | |
| **Model test *p* value** | <0.001 | | | |
| **AIC** | 110433.4 | | | |
| **BIC** | 110619 | | | |

**Table S13. Mixed effects regression model of belief in falsehoods with interaction between story type and average social media use.**

| | Coefficient | Std. error | *P* value | 95% CI |
|---|---|---|---|---|
| **Satire** | -0.072 | 0.028 | 0.010 | -0.127, -0.017 |
| **Education** | -0.025 | 0.010 | 0.016 | -0.046, -0.005 |
| **Political interest** | -0.076 | 0.018 | <0.001 | -0.112, -0.040 |
| **Sex (male = 1)** | -0.056 | 0.029 | 0.059 | -0.113, 0.002 |
| **Age** | -0.001 | 0.001 | 0.235 | -0.003, 0.001 |
| **Party ID (Democrat = 1)** | -0.239 | 0.030 | <0.001 | -0.298, -0.179 |
| **Ingroup-beneficial statement** | 0.103 | 0.012 | <0.001 | 0.080, 0.126 |
| **Ingroup-harmful statement** | -0.236 | 0.011 | <0.001 | -0.258, -0.214 |
| **Faith in Intuition for Facts** | 0.100 | 0.024 | <0.001 | 0.054, 0.146 |
| **Need for Evidence** | -0.019 | 0.026 | 0.463 | -0.069, 0.031 |
| **Truth is Political** | 0.086 | 0.020 | <0.001 | 0.046, 0.125 |
| **Conspiracy mentality** | 0.148 | 0.024 | <0.001 | 0.101, 0.195 |
| **Average online news use** | 0.004 | 0.005 | 0.399 | -0.006, 0.015 |
| **Average offline news use** | 0.027 | 0.012 | 0.022 | 0.004, 0.049 |
| **Average social media use** | 0.023 | 0.015 | 0.127 | -0.007, 0.053 |
| **Average social media use x Satire** | 0.001 | 0.012 | 0.898 | -0.021, 0.024 |
| **Facebook engagement (log)** | 0.035 | 0.004 | <0.001 | 0.028, 0.042 |
| **Intercept** | | | <0.001 | 1.117, 1.800 |
| **Number of observations** | 50926.000 | | | |
| **Number of clusters** | 480.000 | | | |
| $\chi^2$ | 1164.660 | | | |
| **Model test *p* value** | <0.001 | | | |
| **AIC** | 110434.1 | | | |
| **BIC** | 110619.7 | | | |

**Table S14. Mixed effects regression model of belief in falsehoods with interaction between story type and social media engagement.**

| | Coefficient | Std. error | *P* value | 95% CI |
|---|---|---|---|---|
| **Satire** | -0.261 | 0.084 | 0.002 | -0.426, -0.096 |
| **Education** | -0.025 | 0.010 | 0.016 | -0.046, -0.005 |
| **Political interest** | -0.076 | 0.018 | <0.001 | -0.112, -0.040 |
| **Sex (male = 1)** | -0.056 | 0.029 | 0.059 | -0.113, 0.002 |
| **Age** | -0.001 | 0.001 | 0.235 | -0.003, 0.001 |
| **Party ID (Democrat = 1)** | -0.238 | 0.030 | <0.001 | -0.298, -0.179 |
| **Ingroup-beneficial statement** | 0.104 | 0.012 | <0.001 | 0.080, 0.127 |
| **Ingroup-harmful statement** | -0.236 | 0.011 | <0.001 | -0.258, -0.214 |
| **Faith in Intuition for Facts** | 0.100 | 0.024 | <0.001 | 0.054, 0.146 |
| **Need for Evidence** | -0.019 | 0.026 | 0.464 | -0.069, 0.031 |
| **Truth is Political** | 0.086 | 0.020 | <0.001 | 0.046, 0.125 |
| **Conspiracy mentality** | 0.148 | 0.024 | <0.001 | 0.101, 0.195 |
| **Average online news use** | 0.004 | 0.005 | 0.403 | -0.006, 0.015 |
| **Average offline news use** | 0.027 | 0.012 | 0.022 | 0.004, 0.049 |
| **Average social media use** | 0.024 | 0.014 | 0.099 | -0.004, 0.052 |
| **Facebook engagement (log)** | 0.031 | 0.004 | <0.001 | 0.023, 0.039 |
| **Facebook engagement (log) x Satire** | 0.017 | 0.007 | 0.018 | 0.003, 0.031 |
| **Intercept** | | | <0.001 | 1.158, 1.846 |
| **Number of observations** | 50926.000 | | | |
| **Number of clusters** | 480.000 | | | |
| $\chi^2$ | 1165.873 | | | |
| **Model test *p* value** | <0.001 | | | |
| **AIC** | 110429.2 | | | |
| **BIC** | 110614.8 | | | |

**Supplementary Figures**

**Fig S1. Estimated strength of belief by faith in intuition and source of falsehood.**

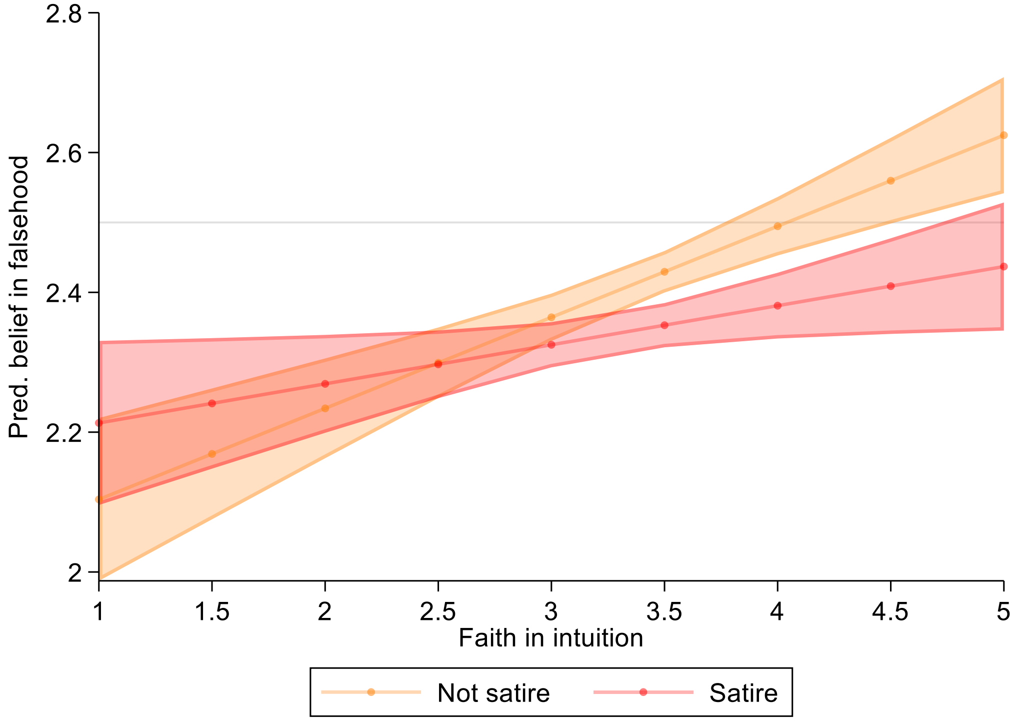


Points correspond to point estimates with lines denoting 95% confidence intervals. Thin grey line indicates scale midpoint.

**Fig S2. Estimated strength of belief by perception that truth is political and source of falsehood.**

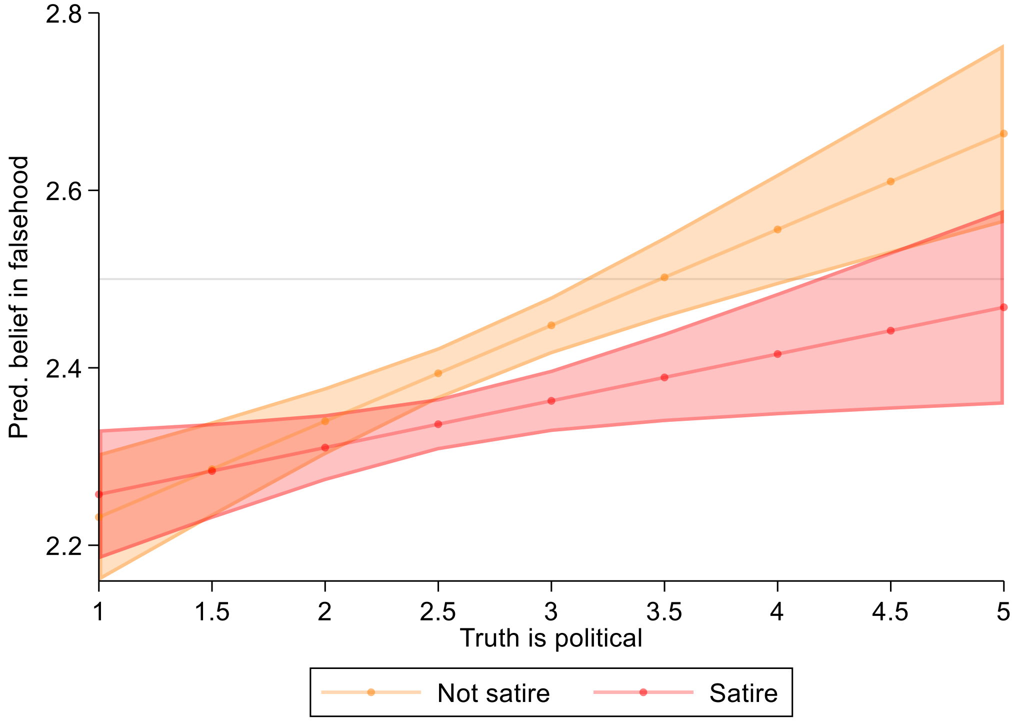


Points correspond to point estimates with lines denoting 95% confidence intervals. Thin grey line indicates scale midpoint.

**Fig S3. Estimated strength of belief by conspiracy mentality and source of falsehood.**

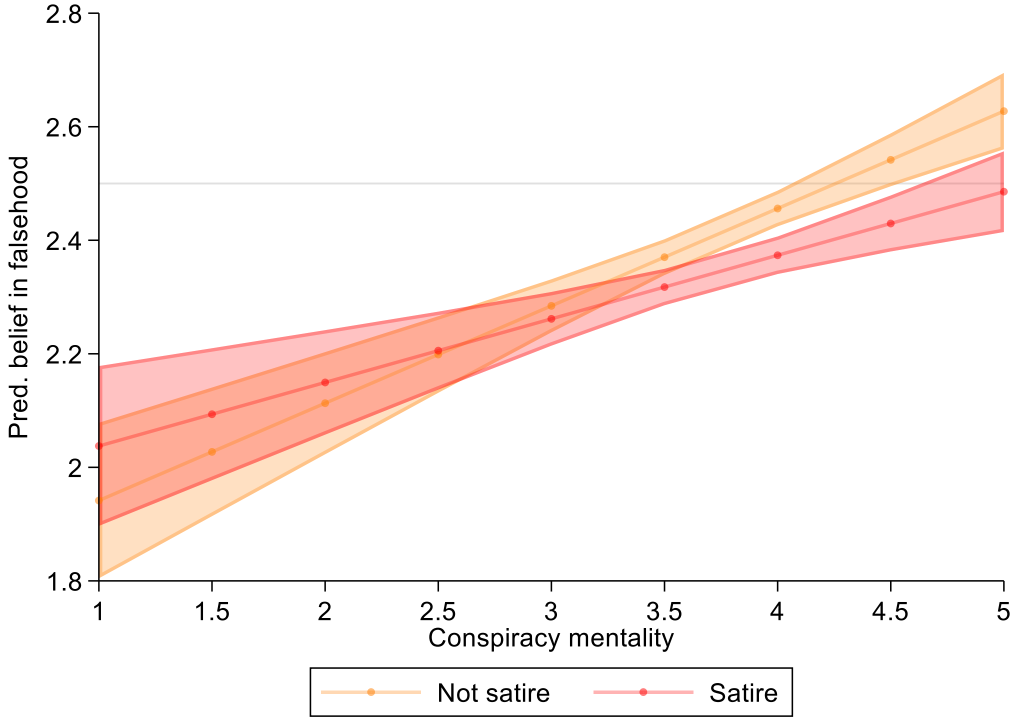


Points correspond to point estimates with lines denoting 95% confidence intervals. Thin grey line indicates scale midpoint.

**Fig S4. Estimated strength of belief by average online news use and source of falsehood.**

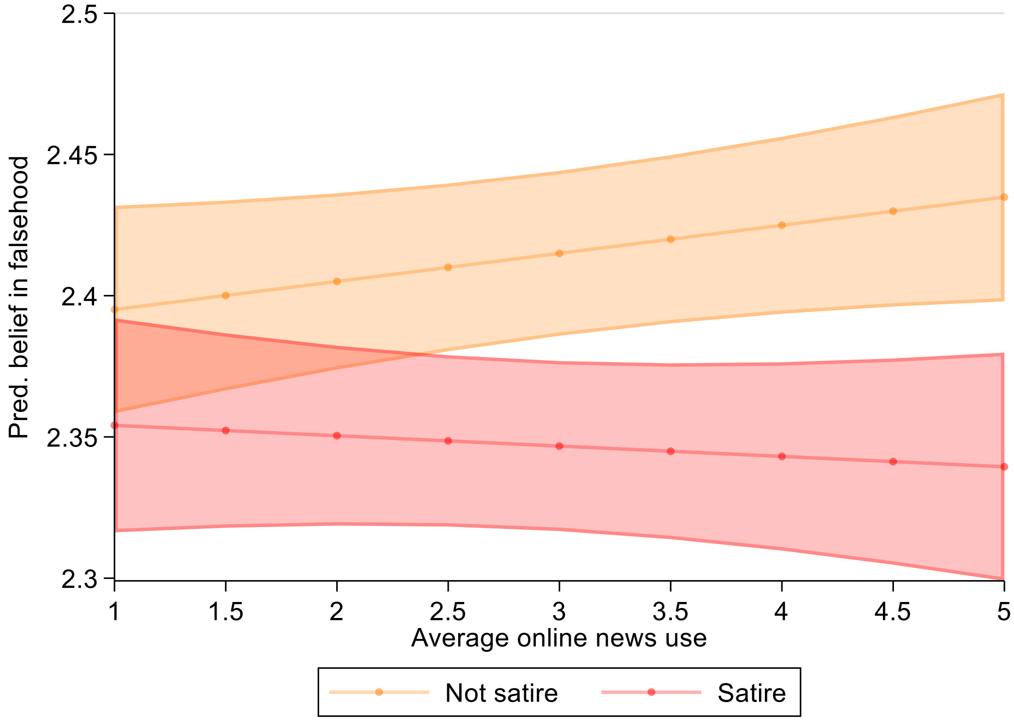


Points correspond to point estimates with lines denoting 95% confidence intervals. Thin grey line indicates scale midpoint.
